# Automatic Construction of Predictive Neuron Models through Large Scale Assimilation of Electrophysiological Data

**DOI:** 10.1038/srep32749

**Published:** 2016-09-08

**Authors:** Alain Nogaret, C. Daniel Meliza, Daniel Margoliash, Henry D. I. Abarbanel

**Affiliations:** 1 Department of Physics, University of Bath, Bath BA2 7AY, UK; 2 Department of Psychology, University of Virginia, Charlottesville, VA 22904, USA; 3 Department of Organismal Biology and Anatomy, University of Chicago, Chicago, IL 60637, USA; 4 Department of Physics, University of California San Diego, La Jolla, CA 92093, USA; 5 Scripps Institution for Oceanography, Marine Physical Laboratory, La Jolla, CA 92093, USA.

## Abstract

We report on the construction of neuron models by assimilating electrophysiological data with large-scale constrained nonlinear optimization. The method implements interior point line parameter search to determine parameters from the responses to intracellular current injections of zebra finch HVC neurons. We incorporated these parameters into a nine ionic channel conductance model to obtain completed models which we then use to predict the state of the neuron under arbitrary current stimulation. Each model was validated by successfully predicting the dynamics of the membrane potential induced by 20–50 different current protocols. The dispersion of parameters extracted from different assimilation windows was studied. Differences in constraints from current protocols, stochastic variability in neuron output, and noise behave as a residual temperature which broadens the global minimum of the objective function to an ellipsoid domain whose principal axes follow an exponentially decaying distribution. The maximum likelihood expectation of extracted parameters was found to provide an excellent approximation of the global minimum and yields highly consistent kinetics for both neurons studied. Large scale assimilation absorbs the intrinsic variability of electrophysiological data over wide assimilation windows. It builds models in an automatic manner treating all data as equal quantities and requiring minimal additional insight.

The extraction of information hidden from observation is essential to understanding and modelling complex nonlinear systems^1^. This approach is relevant to the construction of models of biological neurons which relate unobserved ionic transport at the molecular level to real time macroscopic observations of the membrane voltage. While remaining largely inaccessible to experiment, the dynamics of numerous voltage-gated ionic channels acting in parallel through complex nonlinear relationships plays a critical role in shaping the membrane potential^2^. Statistical inference methods can therefore be valuable for extracting the nonlinear parameters controlling ion channel dynamics when coupled with accurate ionic conductance models of the neurons. This is part of the larger effort to understand the higher functions of neural systems through simulation^3^. Several data assimilation procedures including random parameter search^4^,^5^,^6^,^7^,^8^, evolutionary^9^ and genetic algorithms^10^,^11^,^12^,^13^,^14^,^15^, gradient descent methods^16^ and simulated annealing^17^ have used semi-empirical Hodgkin-Huxley models^2^ to fit linear parameters such as the maximal conductances of ion channels while using tabulated values for the nonlinear parameters such as gate thresholds and time delays^18^,^19^,^20^. Of potential utility would be to identify nonlinear optimization algorithms for data assimilation that would further improve model accuracy by extracting all parameters from experimental data. Such approaches are underpinned by Takens’ embedding theorem that states that all information required to constrain the model is contained in the observation of one state variable – the membrane voltage - over a finite time window^21^,^22^. Two high dimensional nonlinear parameter search algorithms have been tested on conductance models. Vavoulis *et al.*^23^ have estimated *Lymnaea* motoneuron parameters by performing time series analysis with Kitagawa’s self-organizing state space approach^24^,^25^. Meliza *et al.*^26^ have built models of neurons from the zebra finch forebrain nucleus HVC using interior point optimization^27^,^28^.

Here we demonstrate a large-scale nonlinear optimization method for building complex neuron models. We describe methodology that allowed us to transfer information from observations of the membrane voltage to biologically relevant models. Meliza *et al.*^26^ previously studied a large population of neurons using a benchmark electrophysiological protocol which was required to categorize different classes of neurons. The present work instead investigates the responses of individual neurons to a wide range of current protocols. The wide dynamic range covered by these protocols allows us to validate the constructed models both in terms of their predictive power and the plausibility of the extracted parameters.

The method implements Interior Point OPTimization line parameter search (IPOPT)^27^,^29^. We used this approach to assimilate large sets of electrophysiological data from two HVC neurons and inferred the 71 parameters of a multichannel conductance model. Following assimilation, the parameter solutions were incorporated into the conductance model to obtain a completed model for each neuron. Completed models were then used to predict the state of HVC neurons by forward integration of time series current data. IPOPT minimizes a least square function measuring misfit between observations of the membrane voltage and the corresponding state variable in the conductance model. Minimization is subject to both inequality constraints that specify the search interval of model parameters and equality constraints that specify the rate of change of state variables prescribed by the nonlinear conductance model. Interior point optimization replaces inequality constraints with logarithmic barriers bounding the search domain. These initially provide a convex surface ensuring smooth convergence of the parameter search. The accuracy of the solution is improved through subsequent iterations whilst the barrier decreases. The IPOPT algorithm implements a line parameter search filter which, under mild assumptions on the model, is designed to prune spurious solutions to retain the true solution at the global minimum of the objective function^30^.

The completed models were validated by successfully predicting the outcome of experiments that subjected individual neurons to 20–50 current injection protocols some of which had complex dynamics that included both steps and chaotic currents. By comparing the experimental membrane voltages to predictions, we identified the criteria that the assimilation current protocol needs to fulfill in order to effectively constrain the model parameters. The inferred initial state of the neuron was correctly found to be the steady state as expected for an isolated neuron with no prior external stimulation. The biological relevance and uniqueness of parameter solutions rests on the accuracy with which the model describes microscopic gating mechanisms. We addressed this by reformulating our conductance model to comply with the dual needs of biological accuracy and mathematical stability required by constrained optimization. We performed a statistical study of the parameters extracted from different epochs. This identified the recovery time constants as the parameters with the highest degree of functional overlap (“sloppiness”)^31^,^32^. Extracted parameters were found to lie on an ellipsoid surface whose principal axes have an exponentially decaying distribution. A much improved estimate of the global minimum was obtained by calculating the maximum likelihood expectation (MLE) of extracted parameters. The method inferred similar values for the kinetics of the two neurons studied.

Our large scale assimilation method offers many advantages over existing methods. In our approach, all data are assimilated as equal quantities. Completed models were constructed automatically from a generic conductance model that had been previously identified based on biological experiments that relied on pharmacological manipulations to positively identify relevant classes of ionic currents^33^. Automatically generated predictions were in excellent agreement with experiment over virtually all epochs. Biological intuition is minimal and limited to the choice of the parameter search intervals. The assimilation of large scale data absorbs the intrinsic fluctuations of biological neurons to make predictions with accuracy sufficient to evidence trial-to-trial fluctuations in neuron behaviour.

## Results

### Nonlinear optimization framework

A standard brain slice preparation that included HVC was used for *in-vitro* intracellular recordings of HVC neurons. Neurons were stimulated with a wide range of intracellular injections of current protocols in a series of measurement epochs *e* (Supplementary, Fig. S1). We used these data to construct a database of epochs recording the membrane voltage 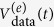 and current stimulation 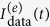. A data assimilation window of duration *T* was selected from the epoch most suitable for assimilation (Epoch 0). The assimilation window contained *N* + 1 observations of the membrane voltage at times *t*_*i*_ = *iT*/*N*, *i* = 0, 1 … *N*. Optimization was performed by minimizing a cost function which measures the discrepancies between the experimental voltage and the model output *V*:


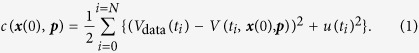


We represent the state of the neuron by a time dependent vector ***x***(*t*) which has *L* vector components. The first component tracks the membrane voltage *x*_1_(*t*) ≡ *V*(*t*) and the others {*x*_*l*_(*t*)}_*l*=2,3…*L*_ ≡ {*m*(*t*), *h*(*t*)} the state of activation and inactivation gates of individual ion channels (Table 1). Overall our conductance model incorporates 9 ion channels giving *L* = 12 state variables. The model also has *K* parameter constants which contain information about the ion channel conductances, gate thresholds, gate time delays and electric parameters of the neuron membrane such as capacitance and reversal potentials. These parameters, listed in Table 2, are stored in the *K *= 71 vector-components of vector ***p***. The objective of nonlinear optimization is to find the parameters p^*^ and initial conditions x^*^(0) which minimize the cost function. The function *u*(*t*) in Eq. 1 was used to smooth convergence of the parameter search by eliminating the occurrence of positive conditional Lyapunov exponents^34^. *u*(*t*) was defined as a control variable of the assimilation procedure which vanishes as the parameter search approaches the global minimum.

The state variables obey the nonlinear rate equations:


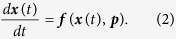


These equalities place *L* constraints ***C***(***X***) = **0** at each point of the assimilation window where ***X*** ≡ {***p***, ***x***(0)} are the generalized optimization parameters including the initial conditions. We linearized Eq. 2 at each node *t*_*i*_ using Simpson’s method^35^ to obtain *L*(*N* + 1) equality constraints. A second set of constraints was specified by setting the minimum and maximum boundaries of the parameter search interval: ***p***_*L*_ ≤ ***p*** ≤ ***p***_*U*_. Minimizing the cost function in the presence of both types of constraints was done seeking the extrema of a Lagrangian function 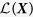 that included both Lagrange multiplier terms for the equality constraints and logarithmic barrier terms for the inequality constraints^27^,^29^. Logarithmic barriers formulated the inequality constrained optimization problem into an equality constrained convex problem which could be solved by Newton’s method. The extrema of the Lagrangian were obtained by solving 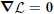 iteratively reducing the barrier height at each iteration. The convex nature of the problem means that the Hessian 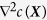 and Jacobian 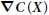 used in Newton’s method are smooth at boundaries ensuring that the parameter search converges to the global minimum even for poor choices of starting points in parameter space. Interior point optimization is well suited to neuron model building because it accommodates the very large number of constraints (>10^6^) needed to assimilate complex electrophysiological data.

### Conductance model

We reformulated our conductance model to comply with the stability requirements of the inverse problem while retaining the level of detail provided by the Boltzmann functions used for fitting biological neurons^2^,^36^,^37^. The need for the Hessian to be continuous and well-behaved called for rate functions ***f*** to be doubly differentiable over the [−120 mV, +50 mV] range of variation of the membrane voltage. This ruled out the use of functions defined in parts which are convenient fitting functions in electrophysiology^36^,^37^. In addition, the model had to describe the kinetics with a minimum of parameters to minimize functional overlap between them and warrant uniqueness of the solution.

The first equation in Eq. 2 is the current conservation equation which governs the rate of change of the membrane voltage:


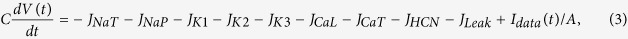


where *C* is the membrane capacitance per unit area, *J*_*α*_, *α* ≡ {*NaT*, *NaP*, *K*1, *K*2, *K*3, *CaL*, *CaT*, *HCN*, and *Leak*} are the current densities of each ion channel, and *A* is the surface area of the neuron membrane through which the external current is injected. A regularization term *u*(*t*)[*V*(*t*) − *V*_data_(*t*)] was added to the right hand side of Eq. 3 to perform assimilation. This term stabilizes convergence of the parameter search by smoothing irregularities in the objective function which are likely to appear when the logarithmic barrier decreases^34^. The ionic currents are chosen based on prior biological knowledge. Each one has a unique mathematical form (Table 1) which facilitates the assignment of parameters and minimizes model degeneracy. An advantage of our data assimilation method is that it does not require biological intuition to assume which ion channel might or might not be present in the experiment because the IPOPT filter automatically assigns a null value to the conductances of the missing channels. A generic conductance model may therefore be used to fit the behavior of different categories of neurons.

The remaining rate equations in the differential system of Eq. 2 describe the first order dynamics of gate variables *m* and *h*:


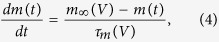



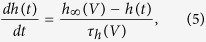


for all 9 ion channels. The steady state activation curves of gate variables and time delays were:


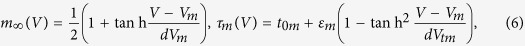



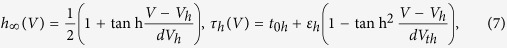


where *V*_*m*_ and *V*_*h*_ are the gate activation and inactivation thresholds, *dV*_*m*_ and *dV*_*h*_ give the slope of the transition from open to closed state, *t*_0*m*_ (resp. *t*_0*h*_) is the recovery time of the activation (resp. inactivation) variable in the closed state while *t*_0*m*_ + *ε*_*m*_ (resp. *t*_0*h*_ + *ε*_*h*_) is the recovery time in the fully open state. The inactivation kinetics of the *K*2 and *CaT* currents has two distinct recovery rates above and below a transition voltage which require different mathematical descriptions^36^,^37^. Below *V*_*h*_ + *δ*_*h*_, the recovery time of *K*2 follows the bell-shaped dependence of Eq. 7. Above the threshold, it becomes independent of the membrane voltage^36^. We describe this two-part behavior with the following equation valid for any membrane voltage:


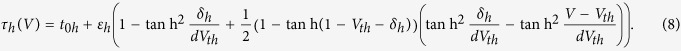


The inactivation kinetics of the *CaT* channel is similarly controlled by two recovery times giving a bi-exponential dependence^36^. The two part exponential is combined into a single equation:


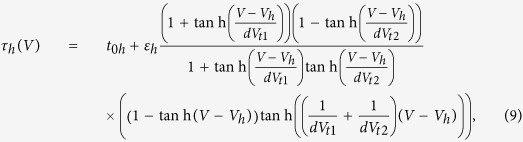


where parameters *dV*_1*t*_ and *dV*_2*t*_ define the recovery times above and below the voltage threshold *V*_*h*_. Our model equations Eqs 6, 7, 8, 9 were validated by fitting successfully the experimental activation functions of thalamocortical neurons (Supplementary Fig. S2).

### Model predictions

**Figure 1:**
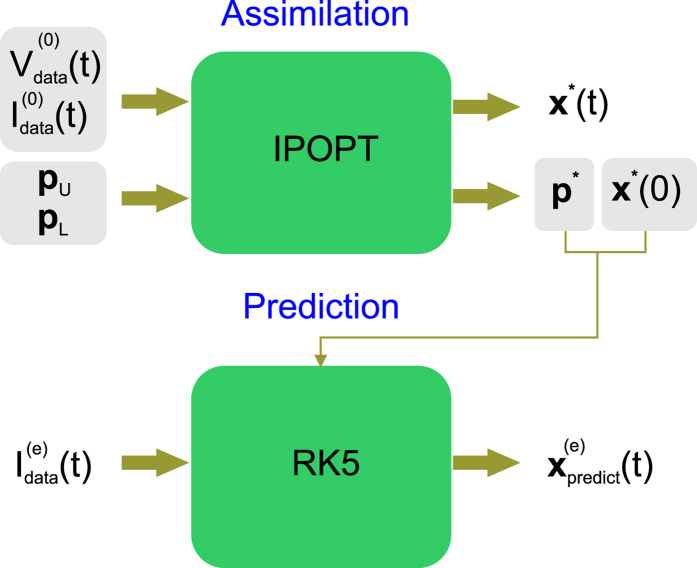
Assimilation-prediction method. The injected current waveform 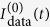 and resultant time series membrane voltage 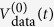 of the epoch to assimilate (Epoch 0) are input into the nonlinear optimization filter (IPOPT). These provide the equality constraints. The user specifies the inequality constraints by choosing the upper and lower boundaries of the parameter search intervals, ***p***_*L*_ and ***p***_*U*_. IPOPT outputs the state variables x^*^(*t*) solution of the minimization problem at each point of the assimilation window, together with the parameter solutions p^*^. The extracted parameters p^*^ are inserted in the model equations to construct the *completed* model. The state of the neuron ***x***_predict_(*t*) is predicted by integrating the current protocol 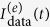 forward from initial conditions x^*^(0) using a fifth order, adaptive step size, Runge-Kutta solver (RK5). The model is validated by comparing the predicted membrane voltage 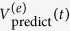 with the voltage 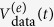 recorded in Epoch *e*.

The assimilated neuron models were primarily validated by testing their ability to predict multiple epochs implementing a wide range of current protocols (see methods). To this end, we constructed the fully automated assimilation-prediction procedure depicted in Fig. 1. In the first stage, the inputs of IPOPT are the electrophysiological recordings 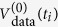 and 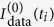, *i* = 0, 1 … *N* chosen to assimilate data from Epoch 0, and the boundaries of the parameter search intervals, ***p***_*L*_ and ***p***_*U*_. IPOPT outputs the state vector that minimizes the objective function at each point of the assimilation window x^*^(*t*_*i*_), *i* = 0, 1 … *N* and the parameter vector solution p^*^. In the second stage, the p^*^ were inserted into the model equations Eqs 6, 7, 8, 9 to obtain the *completed model*. This model was then used to predict the state of this neuron 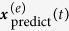 by forward integrating the experimental current protocol of another epoch 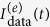. By default, the initial conditions ***x***(0) at the start of integration were obtained from data assimilation (see below). Although the system of equations in Eq. 2 is not believed to be chaotic, the multiplicity of recovery times arising from 9 ion channels induces system stiffness and reduces tolerance to integration error. It was therefore necessary to implement forward integration using adaptive step size fifth order Runge-Kutta (RK5) to achieve the required level of accuracy^38^.

**Figure 2:**
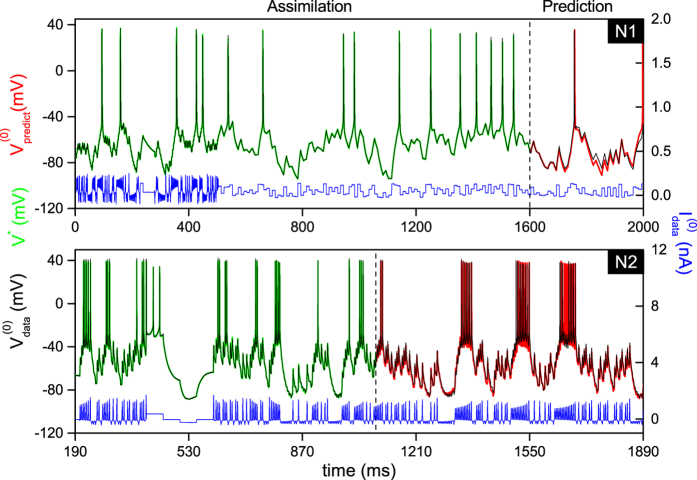
Membrane voltage oscillations used to extract the model parameters of two HVC neurons (Epoch 0). The experimental voltage 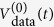 (black line) was recorded under current clamp stimulation by current waveform 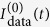 (blue line). N1 is a putative RA-projecting neuron (top panel) and N2 is a X-projecting neuron (bottom panel). The assimilation window used to extract the parameters spans the interval [0–1600 ms] for N1 and [190 ms–1090 ms] for N2. The membrane voltage solution of the constrained optimization problem is *V* ^*^(*t*) (green line). The membrane voltage predicted by integrating the experimental current waveform is 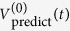 (red line). Details of the oscillations of N2 are plotted in supplementary Figure S7.

Figure 2 shows the two reference epochs which we use to construct the completed models of two representative HVC neurons: a putative RA-projecting neuron (N1) and a putative X-projecting neuron (N2). The current protocol (blue line) induced the oscillations observed in the membrane voltage (black line). Data assimilation was performed over 1600 ms long time interval (N1) and 900 ms (N2) using *N* = 80,000 and *N* = 90,000 mesh points respectively. The mesh size *T*/*N* was chosen to sample potential spikes with ≈100 data points each. The width of the data assimilation window was chosen as a tradeoff between the need to incorporate a statistically meaningful number of spikes and the need to minimize numerical error that accrues when handling larger Jacobian and Hessian matrices. With a constant mesh size, the optimum number of data points was empirically found to be *N* ≈ 100,000.

The state variables x^*^(*t*_*i*_), *i* = 0, 1 … *N* solution of the constrained optimization include the membrane voltage V^*^(*t*_*i*_) (green line, Fig. 2) and the 11 gate variables (Supplementary Figs S5 and S6). The parameter solutions p^*^ are listed in Table 2. The completed models of both N1 and N2 are found to synchronize well to the experimental data. The fitting error was maximum at the site of voltage spikes where |(V ^*^(*t*_*i*_) − *V*_*data*_(*t*_*i*_))/*V*_*data*_(*t*_*i*_)| ≤ 6% (Supplementary Fig. S4). Elsewhere, the fit of subthreshold oscillations produced less than 1% error.

The state of each neuron was predicted beyond the end of the assimilation window by integrating the completed model forward (red curves, Fig. 2). We used the state variables x^*^(*T*) generated by IPOPT at the end of the assimilation window as initial conditions for forward integration. The predicted oscillations of the membrane potential are identical whether forward integration starts from the beginning of the assimilation window or from the end. The predicted timings of voltage spikes, spike shape, spike amplitude and sub-threshold oscillations are in remarkable agreement with the experiment. Occasionally current stimulation near the firing threshold causes a missing or added spike. These discrepancies are likely to be due to spontaneous synaptic activity in the slice rather than from model inaccuracy. This can be seen in Supplementary Fig. S7 (an expanded plot of Fig. 2) where three spikes at *t* = 1653, 1657 and 1662 ms (blue arrows) are missing at the centre of a spike burst but are predicted by the model. Because the amplitude of current stimulation increases progressively from 1640 ms to 1680 ms, N2 could be expected to burst without interruption as it does in the preceding bursts starting at 1340 ms and 1520 ms. This observation underlines an important benefit of large scale data assimilation for integrating stochastic fluctuations in neuron output^39^. By choosing long assimilation windows, stable models may be constructed from imperfect data. Forward integration of Eq. 2 also predicted the dynamics of unobserved gate variables (Supplementary Figs S5 and S6) and ionic currents^26^.

**Figure 3:**
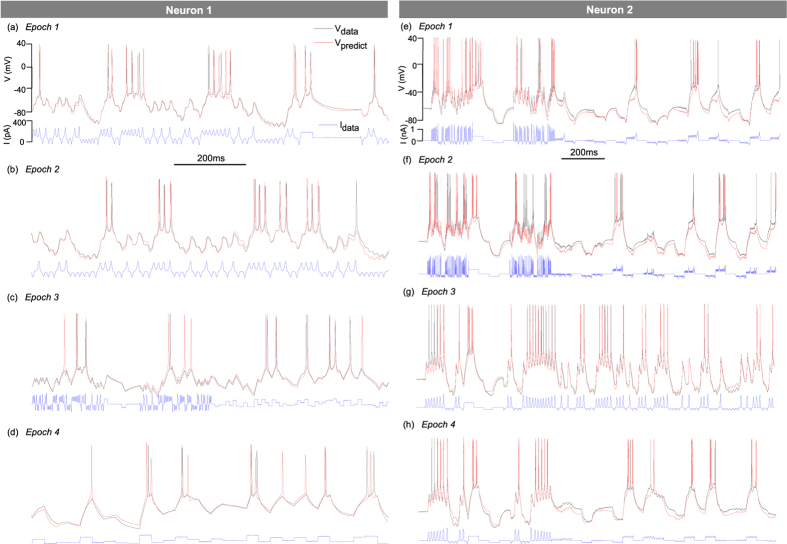
Validation of completed models by comparing predicted membrane voltages (red lines) with experimentally measured voltages (black lines). The current protocols in Epochs 1–4 (blue lines) include a broad range of waveforms and timescales which are distinct from those used to assimilate data. Each current protocol was integrated from the origin onwards to obtain the predicted voltage (red line). The same set of parameters was used to predict all Epochs of N1 (resp. N2).

The completed models are then used to predict the state of a neuron stimulated by different current protocols 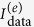 (Epochs *e* = 1–50). The four protocols shown in Fig. 3 test model predictions to current waveforms with different shapes, amplitudes and instantaneous frequencies. Predictions are made from *t* = 0 onwards using initial conditions x^*^(0) computed by IPOPT. Panels (a) and (b) show the predicted voltage of N1 in 1 s long snapshots at the beginning and at the end of a 6 s long epoch through which forward integration has run continuously. The good agreement between the predicted voltage (red line) and the experimental voltage (black line) demonstrates the stability of forward integration over long time intervals. Another factor explaining the good match is the slower rate of oscillations of the stimulating current which is well within the bandwidth of the assimilation protocol (Supplementary Fig. S3). The current stimulation in panels (a),(b) therefore tests time constants of the model which are well constrained by the assimilation protocol. The current protocol of panel (c), in contrast, incorporates oscillations twice as fast as in the assimilation protocol. The predicted voltage occasionally exhibits extra spikes caused by near threshold current oscillations at 90 ms, 430 ms, 450 ms and 950 ms. Panel (d) plots the response to current steps wider than those used in the assimilation protocol (32 ms instead of 10 ms). Although predictions remain good, discrepancies begin to emerge in the response to longer current steps. This can be seen near the end of Epoch 4 where two consecutive current steps effectively form a 64 ms wide pulse. This observation corroborates the fact that tonic spiking elicited by near threshold direct currents is notoriously difficult to reproduce due to stochastic resonance^39^ and oscillations of the subthreshold membrane potential^40^.

We turn next to neuron N2. Both current waveforms in Fig. 3(e,f) are more intricate than the one used in the assimilation step as they mix large amplitude current oscillations, steps, and square pulses modulated by chaotic oscillations. The current oscillations in panel (e) are slower than in the assimilation window (11 ms interspike interval vs 5 ms) whereas they are faster in panel (f) (3 ms interspike interval vs 5 ms). Panels (g) and (h) focus on slowly varying currents. A good agreement is obtained between the predicted and the observed voltages in panel (e) which are driven by relatively slow-varying currents. The response to a rapidly varying current at the start of panel (f) shows several spikes missing from the predicted curve. Prediction here, is complicated by the very short (3 ms) time interval between consecutive current pulses which approaches the width of a voltage spike (~2 ms). The instantaneous frequency of these current oscillations (330 Hz) is above the 200 Hz cut-off frequency of the assimilation protocol (Supplementary Fig. S3). These observations underline the need for wideband assimilation protocols to constraint all time constants of the model.

**Figure 4:**
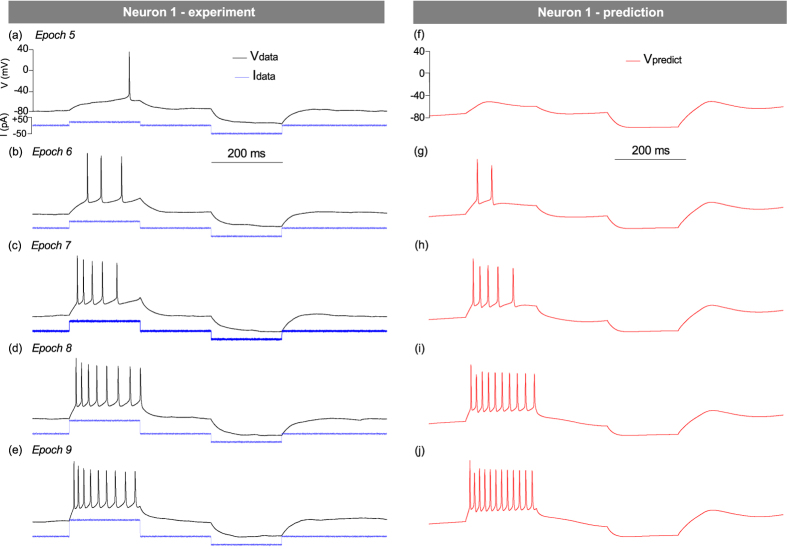
Experimental and theoretical voltage output by Neuron 1 under stimulation by longer current steps. A depolarizing current step is applied between 100 ms and 300 ms. Its amplitude increases from 50 pA (Epoch 5) to 130 pA (Epoch 9) in steps of 20 pA. A constant hyperpolarizing current of −80 pA is applied between 500 ms and 700 ms in all epochs. The predicted voltage (right column) is obtained by integrating the 5 current protocols with the completed model of N1.

We then tested the model ability to predict the excitatory response hence the current threshold of a neuron. The experiment was performed by applying a depolarizing current step of 200 ms duration followed by a hyperpolarizing step (Fig. 4). The amplitude of the depolarizing current was increased through the threshold of the neuron in steps of 20 pA (Panels (a)–(e)). Predictions were made by forward integrating the experimental current protocols of panels (a)–(e) with the completed model of N1. The predicted output in each case is plotted in panels (f)–(j). The model describes the main features of the excitatory response namely, the increase in firing frequency with increasing current stimulation, the correct number spikes per burst (±1 spike), the decay in spike amplitude and widening of inter-spike intervals. The model exaggerates the rebound of the membrane voltage after the release of hyperpolarization. These results show that a single assimilation protocol (Fig. 2) is sufficient to evaluate the firing threshold to a good degree of accuracy: 50 ± 5 pA. Spike bursts evoked by long current steps are difficult to validate through direct comparison with experiment in the manner of Figs 2 and 3 because of the poor reproducibility of real neurons under tonic stimulation^39^,^40^.

### Validation of initial conditions

**Figure 5:**
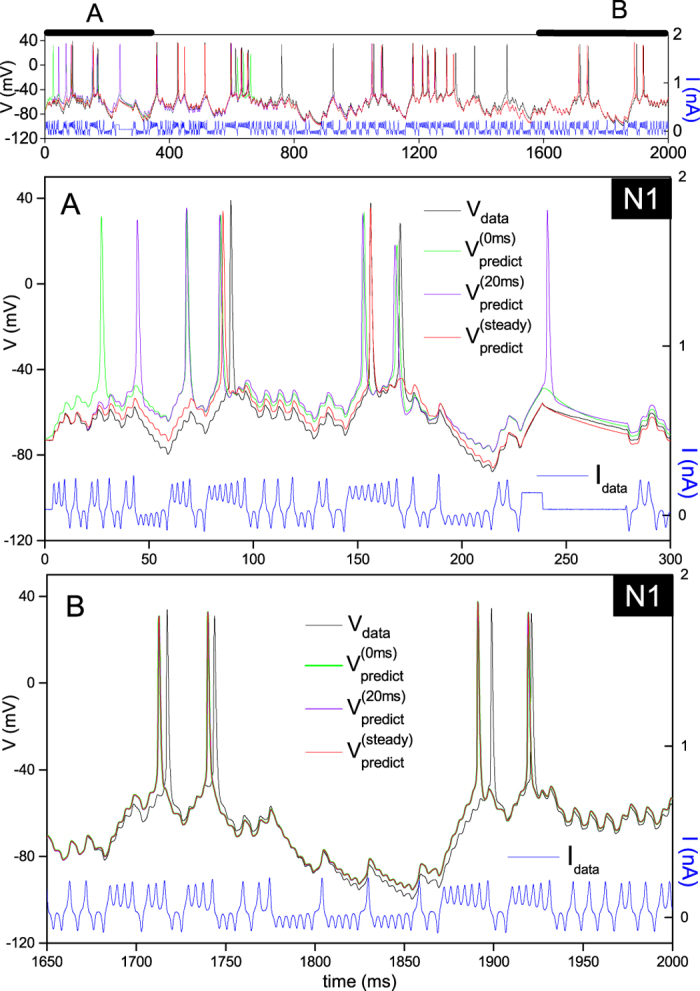
Effect of the choice of initial conditions on model predictions. The initial values of state variables at the beginning of forward integration were obtained by two methods: data assimilation (green and violet lines) and by assuming steady state conditions at the beginning of the epoch (red line). Assimilation of the first 20 ms of data yielded initial conditions at *t* = 0 (green line) and at *t* = 20 ms (violet line). Panel A shows the differences in neuron oscillations induced by different initial conditions over the first 300 ms. These differences however vanish with time. Panel B shows that all predictions become identical near the end of the epoch, irrespective of initial conditions.

The estimation of initial conditions is essential to predicting the state of the neuron, as erroneous starting values often lead to dramatically different behavior over time scales exceeding the relaxation times of the system. The initial state of the neuron ***x***(0) may obtained as a solution of the minimization problem (Eq. 1). Network activity is relatively suppressed in these brain slice recordings, hence it may be reasonably assumed that the initial state of the HVC neuron is the steady state implying 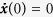. We inserted this condition in Eq. 2 which we solved to obtain the state variables of the neuron in the steady state ***x***_steady_(0). Figure 5 compares the neuron output predicted using initial conditions generated by IPOPT (green and blue lines) and steady state initial conditions (red lines). Results are calculated over a 2 s long protocol (top panel) from which we single out two shorter time intervals at the beginning (panel A) and at the end (panel B) for discussion. Steady state initial conditions (red line) give near perfect predictions in panel A. Initial conditions obtained from assimilation also produce good predictions (to within a few additional spikes). At times larger than the relaxation times of the system, all differences arising from initial conditions vanish and predictions become identical (panel B). This experiment therefore demonstrates that the actual initial state of the neuron is the steady state showing that the neuron is effectively isolated from its environment. We have also verified that the assimilation procedure correctly infers physically meaningful initial conditions.

### Bayesian analysis of extracted parameters

Ideally, the assimilation of different epochs of the same neuron ought to yield identical sets of parameters. In practice, differences in constraints from different current protocols, stochastic variability in neuron output, noise and ill-posedness of the inverse problem introduce uncertainty in the parameter field. We make constructive use of this randomness to obtain additional information about the model. We have extracted *R* sets of parameters 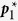, 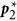 … 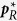 from *R* different epochs to construct a sample of possible values taken by random vector ***P***. Gaussian uncertainty in the probability density implies the solutions of the inverse problem are also Gaussian. This assumes that experimental error is sufficiently small for the forward solutions to cover a small region of parameter space. As a result, the random vector ***P*** follows a normal distribution centered on maximum likelihood expectation P^*^ and characterized by its covariance matrix 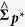
41,42:


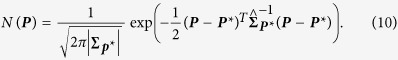


A Taylor expansion of the objective function Eq. 1 with respect to small changes in parameters about the minimum p^*^ yields the data misfit:


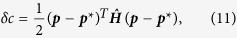


where 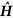 is the Hessian matrix of the data misfit term *δc*. Identification of *δc* with the argument of the exponential in Eq. 10 yields 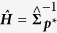. Eq. 11 shows that the surface of constant misfit is a *K*-dimensional ellipsoid in parameter space. The lengths of its principal axes determine the degree of correlation (or sloppiness) between parameters^31^. These are obtained by calculating the square roots of the eigenvalues of 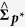: {*ε*_*k*_}, *k* = 1, 2 … *K*. The amplitude of eigenvalues determines the degree of functional overlap between model parameters.

**Figure 6:**
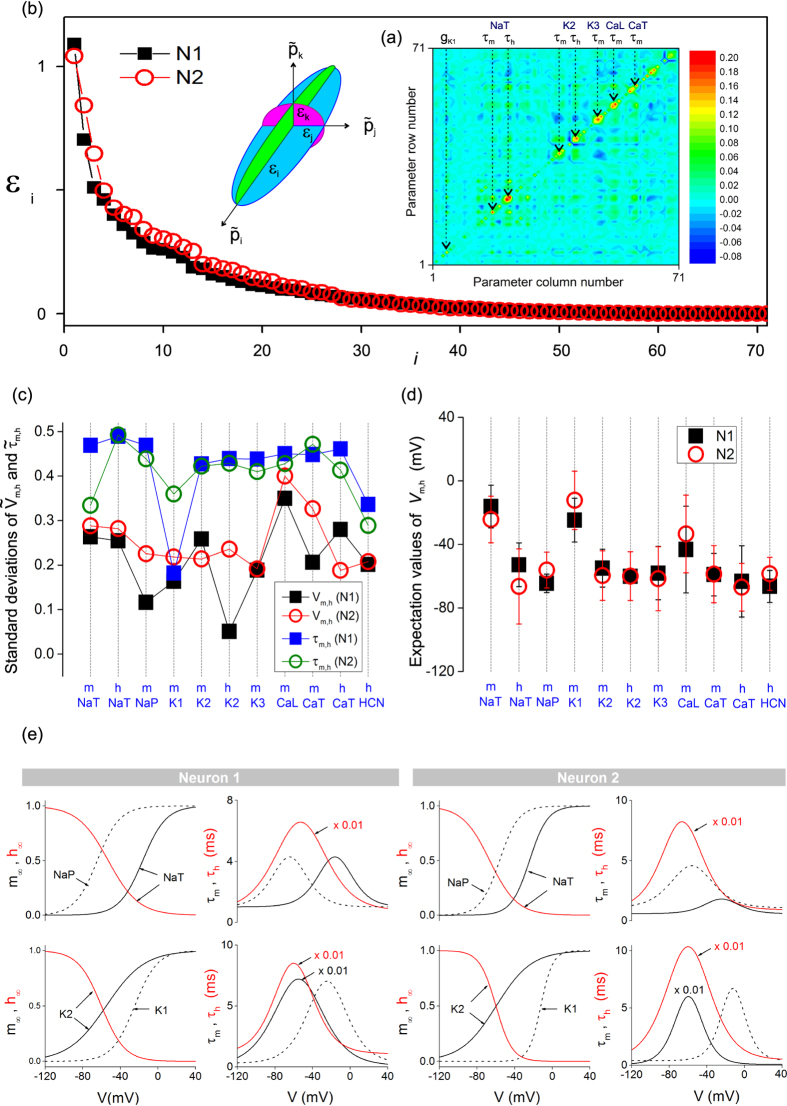
(**a**) Covariance matrix of the random vector 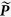 of N1. The dashed arrows indicate the sloppy parameters with the largest off-diagonal matrix elements. (**b**) Spectrum of eigenvalues of the covariance matrix of N1 (black squares) and N2 (red circles) which measure the length of the principal axes of the data misfit ellipsoid (inset). (**c**) Standard deviations of activation and inactivation voltage thresholds and recovery times of neuron N1 (square symbols) and N2 (circles). (**d**) Maximum likelihood expectation values of the activation and inactivation thresholds of N1 (dark squares) and N2 (red circles). The error bars are the standard deviations. (**e**) Stationary values (*m*_∞_, *h*_∞_) and time constants (*τ*_*m*_, *τ*_*h*_) of the activation and inactivation variables of *NaT*, *NaP*, *K*1 and *K*2 ion currents for neurons N1 and N2. These were calculated using the maximum likelihood expectation of parameters.

We have assimilated the data from *R* = 84 time windows for N1 (Supplementary Fig. S8) and likewise for N2 (Supplementary Fig. S9) to obtain forward parameter solutions 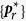, *r* = 1, 2 … 84. This number was chosen because the number forward solutions needed to compute the covariance matrix of the posterior probability density *N*(***P***) had to be greater than the number of parameters (*R* > *L*). We then constructed the covariance matrix of dimensionless parameters 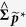 by normalizing individual parameters with respect to their search interval ***p***_*U*_ − ***p***_*L*_. Normalization allows computing meaningful eigenvalues from parameters expressed in different units. The normalized parameters were calculated as: 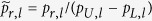, *l* = 1, 2 … *L*, *r* = 1, 2 … *R*. The dimensionless covariance matrix was calculated as:


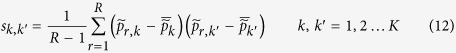


where the 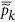 are the mean values of normalised parameters averaged over the statistical sample. The covariance matrix of N1 is mapped in Fig. 6(a). The spectrum of eigenvalues of the N1 and N2 matrices is plotted in Fig. 6(b). Eigenvalues decay exponentially for both N1 and N2 (Supplementary Fig. S10). The majority of these, 57/71, have amplitude less than 10% of the largest eigenvalue which indicates that most parameters are well constrained. To identify the sloppy parameters associated with the larger eigenvalues, we conducted a side by side examination of the covariance matrices of N1 and N2 (Supplementary Fig. S11). The first observation is that finite off-diagonal matrix elements are not random but align in rows and columns which are the same in N1 and N2. Closer examination reveals that sloppy parameters correspond to the recovery times *τ*_*m*_ and *τ*_*h*_ of the *NaT*, *K*2, *K*3, *CaL*, *CaT* channels. The best constrained parameters are the voltage thresholds *V*_*m*_ and *V*_*h*_. This can be seen in Fig. 6(c) which compares the standard deviations of voltage thresholds and gate recovery times. For all ionic gates without exception, the gate recovery time has a larger standard deviation than the voltage threshold. This result may be explained by the choice of more complex equations for recovery times which contain more adjustment parameters that the equations of activation curves.

Experimental error has the same effect as a residual temperature *T* which places a lower limit on the free energy of the system (the cost function *c*). This temperature prevents cooling the system to zero to reach the global minimum. The best that can be hoped is that direct parameter search arrives on the surface of the ellipsoid *δc* = *kT* where *k* = 2(*N*+1)*k*_*B*_*R*Δ*f* is the signal noise entropy, *k*_*B*_ Boltzmann’s constant, *R* the resistance of the neuron and Δ*f* the noise bandwidth. It follows that the random vector ***P*** maps the ellipsoid surface *δc* = *kT* which is centered on its MLE. Assuming that *T* is not so large that the second order expansion Eq. 11 evaluates *δc* with sufficient accuracy, the true global minimum may be obtained by calculating P^*^. We have done this for the gate voltage thresholds *V*_*m*_ and *V*_*h*_ of N1 and N2 and plotted the results in Fig. 6(d). Remarkably, the MLEs give highly consistent threshold values in N1 and N2. In addition, the activation functions and recovery times calculated using MLEs are also in very good agreement in N1 and N2 (Fig. 6(e)).

## Discussion

Our results show that interior point line parameter search successfully addresses several challenges identified as critical to building neuron models^20^,^32^,^43^. Our method extracts both linear and nonlinear parameters from arbitrarily complex time series data. The large scale nature of the problem allows integrating neuron variability and noise over wide assimilation windows to give highly stable completed models. Data assimilation is a fully automatic process that treats all data points as equally important unlike multi-objective methods that select fitting criteria such as spike rate, spike width, spike height, number of spikes in bursts etc^10^,^17^. Each one of our completed models made successful predictions of the state of neurons stimulated by a multitude of current waveforms. We found that discrepancies between predicted and experimental voltages arose when the assimilation protocol loosely constrained the shortest recovery time constants of the model, in particular those of the transient sodium channel. This occurred when the bandwidth of the assimilation protocol was narrower than that of the protocol used for testing predictions. This condition is sufficient but not necessary as there are other factors to overcome, such as noise, to reach the global minimum of the objective function. Recovery times exhibited increased sloppiness relative to other parameters due to the comparatively larger number of adjustment parameters needed to describe complex gate dynamics.

Toth *et al.*^35^ have applied the data assimilation method to model data. They have shown that the solution of well-posed inverse problems involving Hodgkin-Huxley models is single valued. These so called “twin experiments” used the same conductance model to compute the membrane voltage and infer the original model parameters back from it. The challenge of assimilating data from real neurons is that their precise model is unknown. This lack of knowledge was mitigated by analyzing a model of HVC neurons that incorporated ionic currents whose identity had been confirmed previously in biological experiments^33^. Using a model incorporating current types not present in the actual neurons is not problematic since IPOPT assigns near zero values to their ionic conductances. A model lacking an ionic current type expressed in the biological neurons is a more problematic condition. Therefore the possibility of specifying models with numerous ionic currents is an advantageous approach to address the incompleteness of biological knowledge. From a mathematical point of view, the lack of knowledge on the exact equations of the biological neuron makes the problem ill-posed. Simulated annealing experiments have indicated the formation of additional local minima on the free energy surface of conductance models^44^. This possibility was considered in our analysis. However we do not believe local minima to play a role here because the widely different *V*_data_(*t*) observed in N1 and N2 would have produced different free energy surfaces and local minima at different locations in N1 and N2 (see Eq. 1). Parameters having converged to local minima would therefore have produced different covariance matrices and eigenvalue spectra for N1 and N2 which is not what is observed in Supplementary Fig. S11.

Once forward solutions are ascertained to have converged near the global minimum, their maximum likelihood expectation will always provide an even better approximation of the global minimum (Supplementary Fig. S12). The MLE has the additional advantage of cancelling parameter sloppiness. The direct calculation of covariance matrices however carries a high computational cost. A minimum of 71 sets of parameters have to be extracted at a cost of ~50 hours of workstation time per set. Algorithms based on Bayesian uncertainty quantification^41^ may provide an alternative path to estimate these matrices.

The choice of the parameter search interval generally is not critical to convergence as the parameter search generates predictive models even when unrealistically large intervals are used. Setting a too tight parameter range or unreasonable boundaries however is not allowed as the model will not fit the data. Conversely, increasing the 71-dimensional search volume beyond reason, needlessly increases computation time. Our parameter search intervals in Table 2 were chosen to encompass the widest range of biologically plausible values which we further incremented by a safety margin.

Our work makes a number of assumptions. Ligand-gated ion channels are omitted. Although the assimilation method may easily be extended to describe multiple compartments, a single compartment neuron model is found to be adequate to describe currents injected in the soma. The relevant compartment is the axon hillock which leads to spike initiation, has a higher density of Na channels, and is weakly coupled to dendrites. Our nonlinear parameter search is generally very robust having successfully constructed models from 22 out of 26 HVC neurons^26^. In summary, large scale data assimilation combined with Bayesian inference is a very effective method for extracting information on microscopic processes and building the accurate neuron models needed to simulate the higher functions of networks.

## Methods

### Electrophysiology

Songbird neurons have a complex system of ion channels^45^,^46^,^47^,^48^ which make them appropriate for testing multichannel conductance models. Wholecell recordings were made in current clamp mode from HVC brain slices prepared from adult male zebra finches (Taeniopugia guttata). Current clamp measurements were made on male zebra finch HVC neurons, and recordings from 26 neurons were selected for further analysis^26^. Out of these we tentatively identified RA-projecting neurons, X-projecting neurons and interneurons which are broad classes of neurons differing in anatomy^49^,^50^, physiological properties^33^,^51^ and presumably circuit function^52^. Neuron N1 was classified as a RA-projecting neuron based on its lack of adaptation and high firing rate. N2 was identified as a X-projecting neuron based on its lack of adaptation. The current clamp hardware and *in-vitro* solutions are described in Supplementary Fig. S1. Between 20 and 50 different current protocols were applied depending on the duration of the recordings for each neuron. Current protocols were designed with the bandwidth needed to constrain the internal time constants of the neuron (Supplementary, Fig. S3). They were composed of aperiodic oscillations synthesized by the chaotic Lorenz system^53^ mixed with positive and negative current steps calibrated in amplitude to induce depolarization, subthreshold oscillations and hyperpolarization. A flat power spectrum was desirable to give equal weight to the assimilation of the different time constants of the model. The chaotic Lorenz oscillator model fulfils these requirements and is simple to use for synthesizing current waveforms. Some current protocols consisted exclusively of current steps of varying magnitude and duration to characterize the excitatory response of neurons. The input impedance of the amplifier (10^13^ Ω) was orders of magnitude larger than the leakage resistance of any HVC neuron (10^8^–10^10^ Ω) therefore leakage through the measurement system could safely be neglected in Eq. 3. Measurements were taken at a 50 kHz sampling rate over periods lasting 2 s–6 s. The data were eventually linearly interpolated to resolve individual spikes with at least 100 points/spike.

### Numerical analysis

The IPOPT software inputs two text files. The first one specifies the model equations Eqs 3, 4, 5, 6, 7, 8, 9, 10, 11. The second one contains the parameters boundaries ***p***_*L*_ and ***p***_*U*_, the size of the assimilation widow *N*, *T* and the names of the input files containing the data to assimilate. The Jacobian and Hessian matrices **∇*****C*** and ∇^2^*c* are calculated using symbolic differentiation (Sympy) prior to compilation of the executable code. Sparse linear systems are solved by embedding the NAG MAS57 solver in IPOPT. The Goldman-Hodgkin-Katz equation describing calcium dynamics^54^ was computed as a Taylor series expansion, retaining the first 25 terms to obtain the required accuracy over the [−120 mV, +50 mV] range of the membrane voltages. The polynomial was computed efficiently in the Horner form:


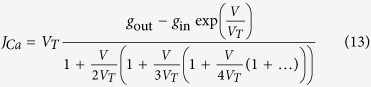


where thermal voltage *V*_*T*_ = 13 mV was set as a constant (*T* = 25 °C). The outer calcium conductance *g*_*out*_ is a parameter of data assimilation. The inner calcium conductance *g*_*in*_ is four orders of magnitude smaller than *g*_out_ and was set at a constant value of 10^−4^ mS.cm^−2^ in the model.

The data files containing the parameter solutions output by IOPT and the current protocol to integrate were read by a custom made C program which performed forward integration by implementing adaptive step fifth order Runge-Kutta method^38^. The regularization term *u*(*t*)[*V*(*t*) − *V*_data_(*t*)] was left out of the completed model being integrated forward. The covariance matrix of the random parameter vector was calculated within a purposely written C program. This matrix being symmetric, its eigenvalues were calculated by applying Jacobi transformations to its elements^38^.

## Additional Information

**How to cite this article**: Nogaret, A. *et al.* Automatic Construction of Predictive Neuron Models through Large Scale Assimilation of Electrophysiological Data. *Sci. Rep.* **6**, 32749; doi: 10.1038/srep32749 (2016).

## Supplementary Material

Supplementary Information

## Figures and Tables

**Table 1:** Ion channels included in the model.

ID	Channel	Current density	Nominal conductance^36^,^37^
**NaT**	Fast and transient Na^+^ current	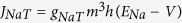	*g*_*NaT*_ = 110 mS.cm^−2^
**NaP**	Persistent Na^+^ current	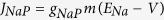	*g*_*NaP*_ = 0.064 mS.cm^−2^
**K1**	Transient depolarization activated K^+^ current	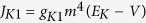	*g*_*K*1_ = 5 mS.cm^−2^
**K2**	Rapidly inactivating K^+^ current (A current)	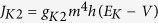	*g*_*K*2_ = 12 mS.cm^−2^
**K3**	Ca^2+^ activated K^+^ current	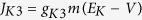	*g*_*K*3_ = 9.1 mS.cm^−2^
**CaL**	High threshold Ca^2+^ current	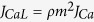	—
**CaT**	Low threshold Ca^2+^ current	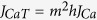	—
**HCN**	Hyperpolarization-activated cation current	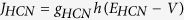	*g*_*HCN*_ = 0.092 mS.cm^−2^
**Leak**	Leakage channels (K & Na)	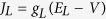	*g*_*L*_ = 0.066 mS.cm^−2^

Parameters include the ion conductances *g*_*α*_, *α* ≡ {*NaT*, *NaP*, *K*1, *K*2, *K*3, *CaL*, *CaT*, *HCN*, and *Leak*}; the sodium and potassium reversal potentials, *E*_*Na*_ and *E*_*K*_; the hyperpolarized-activated cation reversal potential *E*_*HCN*_ = −43 mV^55^. *m* and *h* are the state variables of the activation and inactivation gates. *J*_*Ca*_ is the Calcium current given by the Goldman-Hodgkin-Katz equation^54^.

**Table 2:** Parameters of completed neuron models extracted from Epoch 0.

Ion			Neuron 1		Neuron 2	
	Parameter		*p*_*L*_, *p*_*U*_	p^*^	*p*_*L*_, *p*_*U*_	p^*^
	*C*	*μ*F.cm^−2^	0.3, 4.0	3.80	0.4, 4.0	0.447
	*E*_*Na*_	mV	42, 50	45.57	40, 54	41.55
	*E*_*K*_	mV	−90, −80	−88.29	−95, −60	−95
	*E*_*Leak*_	mV	−110, −40	−64.32	−120, −40	−67.2
	*A*	*μm*^2^	20^2^–50^2^	38.25^2^	20^2^–50^2^	24.8^2^
**NaT**	*g*_*NaT*_	mS.cm^−2^	100, 120	120	70, 130	70
*m*	*V*_*m*_	mV	−49, −27	−37.85	−52, −1	−1.636
	*dV*_*m*_	mV	5, 32	9.16	3, 32	32.0
	*dV*_*tm*_	mV	5, 45	5.0	3, 45	3
	*t*_0*m*_	ms	0.02, 0.7	0.388	0.001, 0.7	0.00647
	*ε*_*m*_	ms	0.012, 7	0.012	0.001, 7	0.06239
*h*	*V*_*h*_	mV	−79, −39	−65.70	−93, −9	−29.35
	*dV*_*h*_	mV	−35, −5	−9.76	−35, −5	−5
	*dV*_*th*_	mV	4, 43	41.67	5, 47	37.89
	*t*_0*h*_	ms	0.02, 90	0.02	0.02, 180	0.02
	*ε*_*h*_	ms	1, 470	4.552	1, 970	13.75
**NaP**	*g*_*NaP*_	mS.cm^−2^	0, 20	0.0062	0, 19	0.9693
*m*	*V*_*m*_	mV	−69, −29	−57.37	−69, −19	−49.80
	*dV*_*m*_	mV	5, 32	9.16	3, 45	38.42
	*dV*_*tm*_	mV	5, 45	5.0	3, 45	45.0
	*t*_0*m*_	ms	0.02, 0.7	0.388	0.001, 7	1.213
	*ε*_*m*_	ms	0.012, 7	0.012	0.001, 7	0.001
**K1**	*g*_*K*1_	mS.cm^−2^	0, 80	0.606	0, 130	130
*m*	*V*_*m*_	mV	−69, −21	−21.0	−39, 25	12.25
	*dV*_*m*_	mV	5, 34	7.561	3, 34	4.860
	*dV*_*tm*_	mV	5, 34	20.11	5, 34	5.0
	*t*_0*m*_	ms	0.01, 5.4	0.34	0.01, 5.4	0.4557
	*ε*_*m*_	ms	0.002, 23	8.556	0.02, 23	10.04
**K2**	*g*_*K*2_	mS.cm^−2^	0, 80	0.430	0, 80	1.128
*m*	*V*_*m*_	mV	−90, −21	−42.71	−90, −17	−83.03
	*dV*_*m*_	mV	5, 48	10.44	5, 48	48.0
	*dV*_*tm*_	mV	5, 48	7.772	5, 48	27.69
	*t*_0*m*_	ms	0.02, 20	1.889	0.02, 20	4.814
	*ε*_*m*_	ms	0.5, 23	23.0	0.5, 143	94.81
*h*	*V*_*h*_	mV	−90, −35	−71.36	−90, −25	−52.68
	*dV*_*h*_	mV	−39, −5	−7.49	−39, −3	−11.05
	*dV*_*th*_	mV	−39, −5	38.99	−39, −5	−25.55
	*t*_0*h*_	ms	0.5, 310	189.7	0.5, 310	15.75
	*ε*_*h*_	ms	0. 910	0.5	0.5, 1910	583.4
	*δ*_*h*_	mV	0, 990	24.35	0, 1990	0
**K3**	*g*_*K*3_	mS.cm^−2^	0, 12	0	0, 6	0.5847
*m*	*V*_*m*_	mV	−25, 30	−24.98	−85, 10	−25, 06
	*dV*_*m*_	mV	10, 65	65	10, 65	53.92
	*dV*_*tm*_	mV	10, 70	70	10, 70	17.87
	*t*_0*m*_	ms	0.01, 55	54.97	0.01, 75	4.066
	*ε*_*m*_	ms	0.1, 455	454.9	0.1, 1955	1955
**CaL**	*g*_*out*_	mS.cm^−2^	0.01, 9	0.119	0.01, 9	0.295
	*ρ*		0.01, 100	0.514	0.01, 100	13.877
*m*	*V*_*m*_	mV	−56, −8	−56.0	−69, −8	−14.21
	*dV*_*m*_	mV	5, 49	20.49	5, 49	49.0
	*dV*_*tm*_	mV	5, 55	12.95	5, 55	21.295
	*t*_0*m*_	ms	0.09, 43.6	32.17	0.09, 73.6	22.20
	*ε*_*m*_	ms	0.2, 295	295	0.2, 1895	1895
**CaT**	V_*m*_	mV	−80, −35	−78.8	−80, −25	−63.32
*m*	*dV*_*m*_	mV	5, 39	39.0	5, 59	59.0
	*dV*_*tm*_	mV	10, 57	53.63	10, 57	10.0
	*t*_0*m*_	ms	0.02, 0.9	0.9	0.02, 0.9	0.02
	*ε*_*m*_	ms	0.5, 97	1.596	0.5, 97	60.63
*h*	*V*_*h*_	mV	−90, −55	−57.04	−85, 25	−72.90
	*dV*_*h*_	mV	−34, −5	−5.0	−44, −3	−29.64
	*dV*_*t*1_	mV	3, 55	55.0	3, 95	43.51
	*dV*_*t*2_	ms	3, 55	55.0	5, 95	95
	*t*_0*h*_	ms	5, 190	50.05	1, 190	11.33
	*ε*_*h*_	mV	0.5, 7000	539.0	0.5, 1600	1600
**HCN**	*g*_*HCN*_	mS.cm^−2^	0, 10	0.013	0, 6	0.2608
*h*	*V*_*h*_	mV	−90, −40	−75.50	−90, −40	−66.92
	*dV*_*h*_	mV	−30, −5	−5.0	−30, −3	−8.142
	*dV*_*th*_	mV	5, 40	40.0	3, 50	4.403
	*t*_0*h*_	ms	0.1, 500	161, 8	0.1, 500	74.741
	*ε*_*h*_	mV	0.1, 5000	0.1	0.1, 500	33.96
**Leak**	*g*_*L*_	mS.cm^−2^	0.01, 0.6	0.0794	0.01, 0.6	0.6
