# Supplementary material for: Automatic Construction of Predictive Neuron Models through Large Scale Assimilation of Electrophysiological Data
Source: Sci Rep. 2016 Sep 8;6:32749. doi: 10.1038/srep32749 (PMC5015021; doi:10.1038/srep32749)
Supplement: Supplementary Information [file srep32749-s1.pdf]

Automatic Construction of Predictive Neuron Models through Large Scale Assimilation of Electrophysiological Data

Alain Nogaret
Department of Physics, University of Bath, Bath, BA2 7AY, UK

C. Daniel Meliza
Department of Psychology, University of Virginia, Charlottesville, VA 22904

Daniel Margoliash
Department of Organismal Biology and Anatomy, University of Chicago, Chicago, IL 60637

Henry D. I. Abarbanel
Department of Physics, University of California San Diego, La Jolla, CA 92093 and Scripps Institute for Oceanography, Marine Physical Laboratory, La Jolla, CA 92093

I. Experimental method

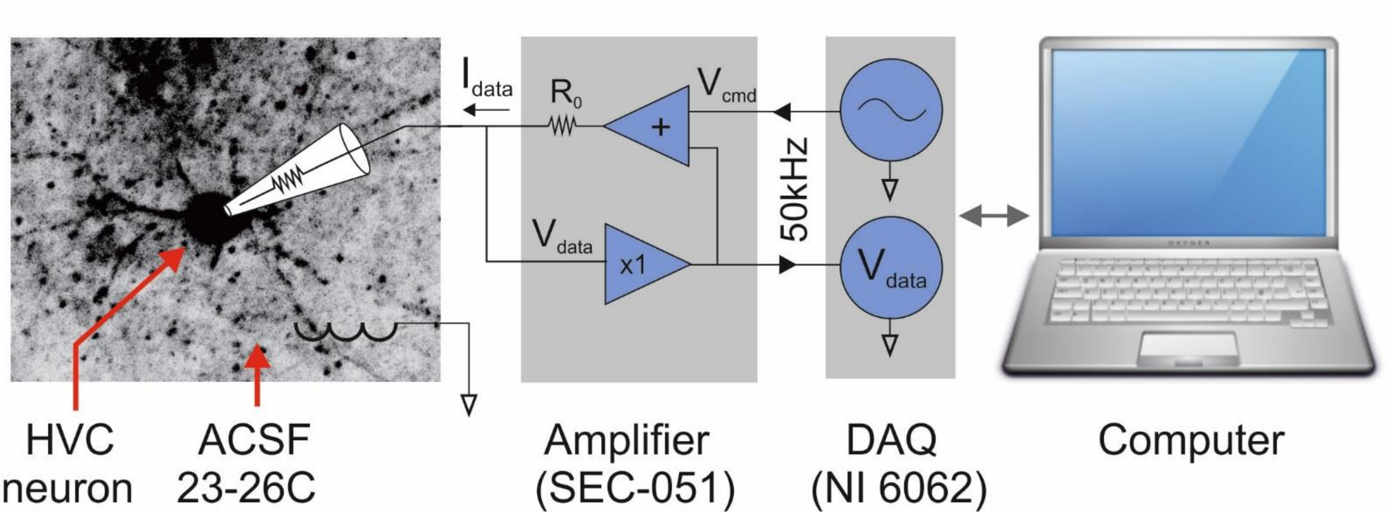

Figure S1: Schematics of current clamp measurements

The computer synthesizes the current protocol applied to the neuron. Its waveform is carried by command voltage $V_{cmd}(t)$ which is added to the observed membrane potential $V_{data}(t)$ by an adder amplifier (+). The amplifier output biases R_0 between $V_{cmd}(t)+V_{data}(t)$ and $V_{data}(t)$ producing an output current $I_{data}(t)=V_{cmd}(t)/R_0$. This current is injected into the neuron via a patch pipette (3-5MΩ) filled with saline solution containing (in mM): 130 K-gluconate, 10 Na-gluconate, 10 HEPES, 4 NaCl, 4 MgATP, 0.3 NaGTP, 10 Na-phosphocreatine, 0.1EGTA (290-310 mOsm, pH 7.2-7.3). *In order to minimize the effect of transmission lines delays in dendrites and axon, the current was injected directly into the soma of the neuron*. The relevant compartment is the axon hillock which leads to spike initiation and has a higher density of Na channels. Neuron slices were immersed in cerebrospinal fluid (ACSF) [Composition (in mM): 123 NaCl, 3 KCL, 1 Na_2POH_4, 26 $NaHCO_3$, 25 D-glucose, 3 $MgSO_4$, 1 CaCl (290-310 mOsm, PH 7.2-7.3)] held at the reference voltage. The neuron membrane voltage was recorded by the DAQ card as $V_{data}(t)$. The sampling frequency of the DAQ card was 50kHz giving a 20μs time interval between consecutive datapoints in the observed I_{data} and V_{data} series.

II. Comparing model activation/inactivation functions used in data assimilation and in Physiology

NaT: *Gate variables*

Model functions used in nonlinear optimization

$$m_\infty(V) = \frac{1}{2}\left(1 + \tanh\left(\frac{V+40}{10}\right)\right)$$

$$h_\infty(V) = \frac{1}{2}\left(1 + \tanh\left(\frac{V+67}{-18}\right)\right)$$

Empirical fitting functions[1]

$$m_\infty(V) = \frac{0.091\dfrac{V+38}{1-\exp\left(-\dfrac{V+38}{5}\right)}}{0.091\dfrac{V+38}{1-\exp\left(-\dfrac{V+38}{5}\right)} - 0.062\dfrac{V+38}{1-\exp\left(\dfrac{V+38}{5}\right)}}$$

$$h_\infty(V) = \frac{0.016\exp\left(-\dfrac{55+V}{15}\right)}{0.016\exp\left(-\dfrac{55+V}{15}\right) + \dfrac{2.07}{\exp\left(\dfrac{17-V}{21}\right)+1}}$$

Recovery times

Model functions

$$\tau_m(V) = 0.26 + 1.09\left(1 - \tanh^2\left(\frac{V+41}{17}\right)\right)$$

$$\tau_h(V) = 1 + 12.9\left(1 - \tanh^2\left(\frac{V+63.5}{24}\right)\right)$$

Empirical fitting functions

$$\tau_m(V) = \frac{1}{0.091\dfrac{V+38}{1-\exp\left(-\dfrac{V+38}{5}\right)} - 0.062\dfrac{V+38}{1-\exp\left(\dfrac{V+38}{5}\right)}}$$

$$\tau_h(V) = \frac{1}{0.016\exp\left(-\dfrac{55+V}{15}\right) + \dfrac{2.07}{\exp\left(\dfrac{17-V}{21}\right)+1}}$$

CaT: *Gate variables*

Model functions

$$m_\infty(V) = \frac{1}{2}\left(1 + \tanh\left(\frac{V+51}{10.5}\right)\right)$$

$$h_\infty(V) = \frac{1}{2}\left(1 + \tanh\left(\frac{V+85}{-6.5}\right)\right)$$

Empirical fitting functions

$$m_\infty(V) = \frac{1}{\left(1+\exp\left(\dfrac{V+57}{-6.2}\right)\right)^2}$$

$$h_\infty(V) = \frac{1}{\left(1+\exp\left(\dfrac{V+81}{4.0}\right)\right)^2}$$

Recovery times

Model functions in nonlinear optimization

$$\tau_m(V) = 0.612 + 13.5662\left(1 - \tanh^2\left(\frac{V+75.8}{26}\right)\right)$$

$$\tau_h(V) = 27 + 297\frac{\left(1+\tanh\left(\dfrac{V+82}{120}\right)\right)\left(1-\tanh\left(\dfrac{V+82}{21.6}\right)\right)}{1+\tanh\left(\dfrac{V+82}{120}\right)\tanh\left(\dfrac{V+82}{21.6}\right)} \times \left[1 - \tanh(V+82))\tanh\left(\left(\frac{1}{82}+\frac{1}{120}\right)(V+82)\right)\right]$$

Empirical fitting functions

$$\tau_m(V) = \frac{1}{\exp\left(\dfrac{V+132}{-16.7}\right) + \exp\left(\dfrac{V+16.8}{18.2}\right)} + 0.612$$

$$\tau_h(V) = \begin{cases} \exp\left(\dfrac{V+467}{66.6}\right) & V < 80mV \\ \exp\left(\dfrac{V+22}{-10.5}\right) + 28 & V > 80mV \end{cases}$$

[1] D.A. McCormick and J.R. Huguenard, *J. Neurophysiol.* **68**, 1384 (1992); *J.Neurophysiol* **68**, 1373 (1992)

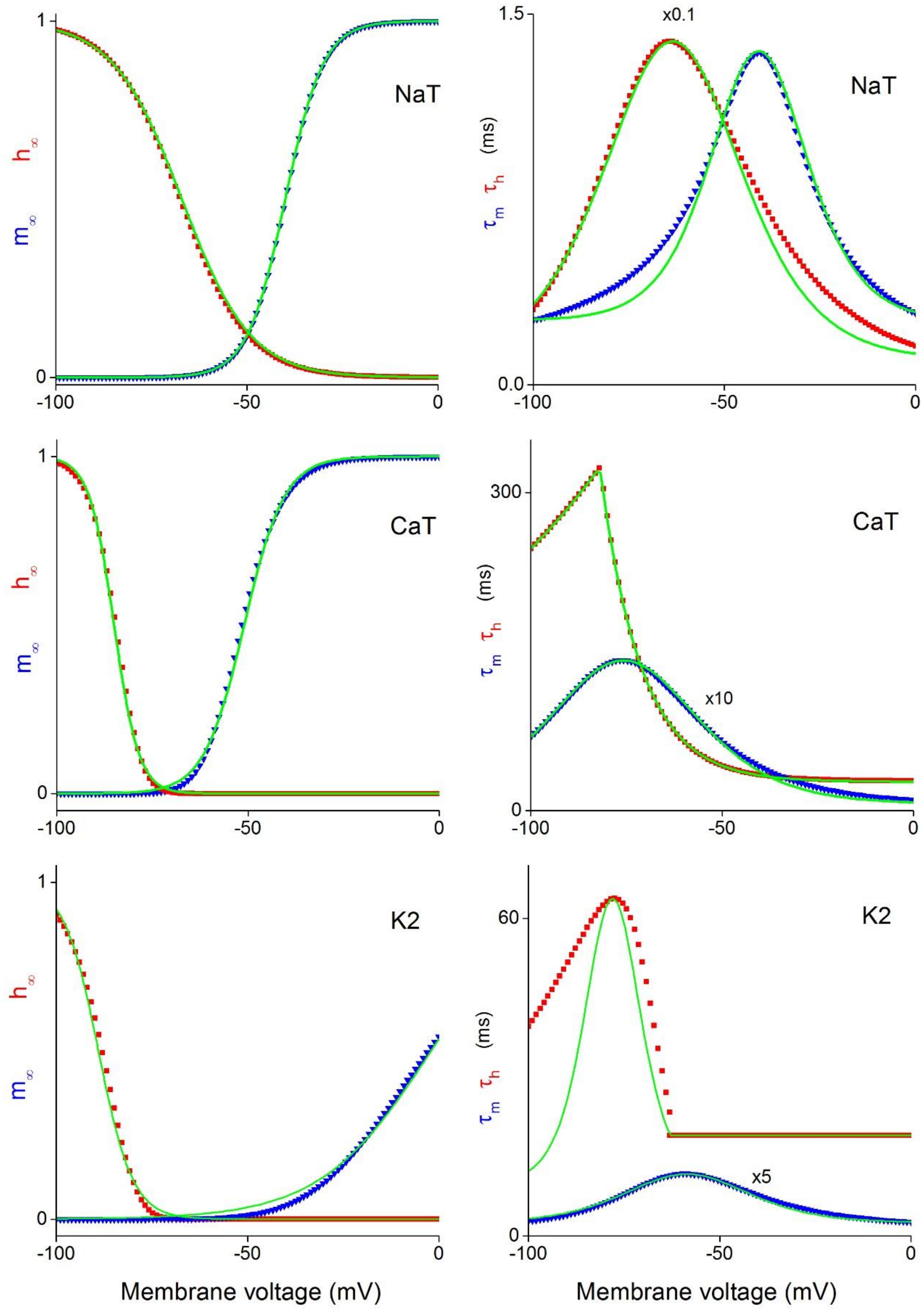

Figure S2: A comparison of the model functions used in nonlinear optimization (Eqs.6-9 of the manuscript, full green lines) with those used to fit the activation (blue symbols) and inactivation (red symbols) of thalamocortical neurons[1]. Both functions and their parameters are given in the above table.

K2: *Gate variables*

Model functions

$$m_\infty(V) = \frac{1}{2}\left(1 + \tanh\left(\frac{V+2}{29}\right)\right)$$

$$h_\infty(V) = \frac{1}{2}\left(1 + \tanh\left(\frac{V+89}{-9}\right)\right)$$

Empirical fitting functions

$$m_\infty(V) = \frac{1}{\left(1+\exp\left(\frac{V+36}{-20}\right)\right)^4}$$

$$h_\infty(V) = \frac{1}{\left(1+\exp\left(\frac{V+78}{6}\right)\right)^4}$$

Recovery times

Model functions

$$\tau_m(V) = 2.5 + 9.2\left(1 - \tanh^2\left(\frac{V+58.6}{22}\right)\right)$$

$$\tau_h(V) = 10 + 53.8 \times \left(\begin{array}{l} 1-\tanh^2\left(\frac{15}{9.724775}\right) \\ +\frac{1}{2}\left(\begin{array}{l}1-\tanh(100(V+63)) \\ \times\left(\tanh^2\left(\frac{15}{9.724775}\right)-\tanh^2\left(\frac{V+78}{9.724775}\right)\right)\end{array}\right)\end{array}\right)$$

Empirical fitting functions

$$\tau_m(V) = \frac{1}{\exp\left(\frac{V+35.8}{19.7}\right)+\exp\left(\frac{V+79.7}{-12.7}\right)} + 0.37$$

$$\tau_h(V) = \begin{cases} \frac{1}{\exp\left(\frac{V+46}{5}\right)+\exp\left(\frac{V+238}{-37.5}\right)} & V < -63mV \\ 19 & V > -63mV \end{cases}$$

III. Frequency spectrum of the current protocols used to assimilate models of N1 and N2

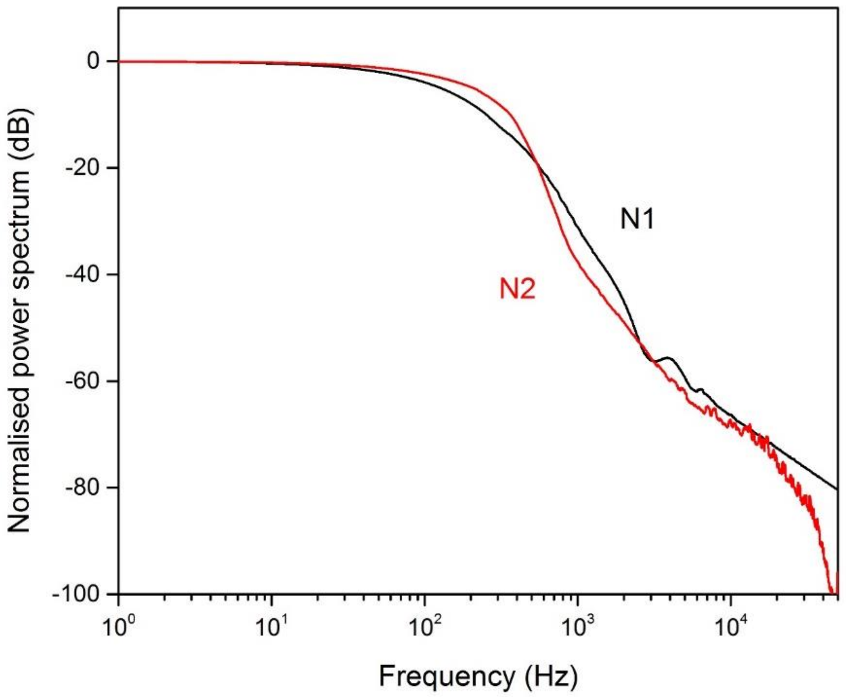

Figure S3: Power spectrum of the current protocols used to extract the parameters of N1 and N2. The low frequency limit (1Hz) corresponds to the width of the assimilation window (1s). The high frequency limit (50kHz) is the sampling frequency of the data. Both current protocols exhibit a low pass spectrum with a cut off frequency of 200Hz. Hence the predictive power of models constructed by assimilating these data is expected to decrease for currents protocols varying on very short timescales, smaller than 5ms - see Fig.3(f).

IV. Estimation of fitting error

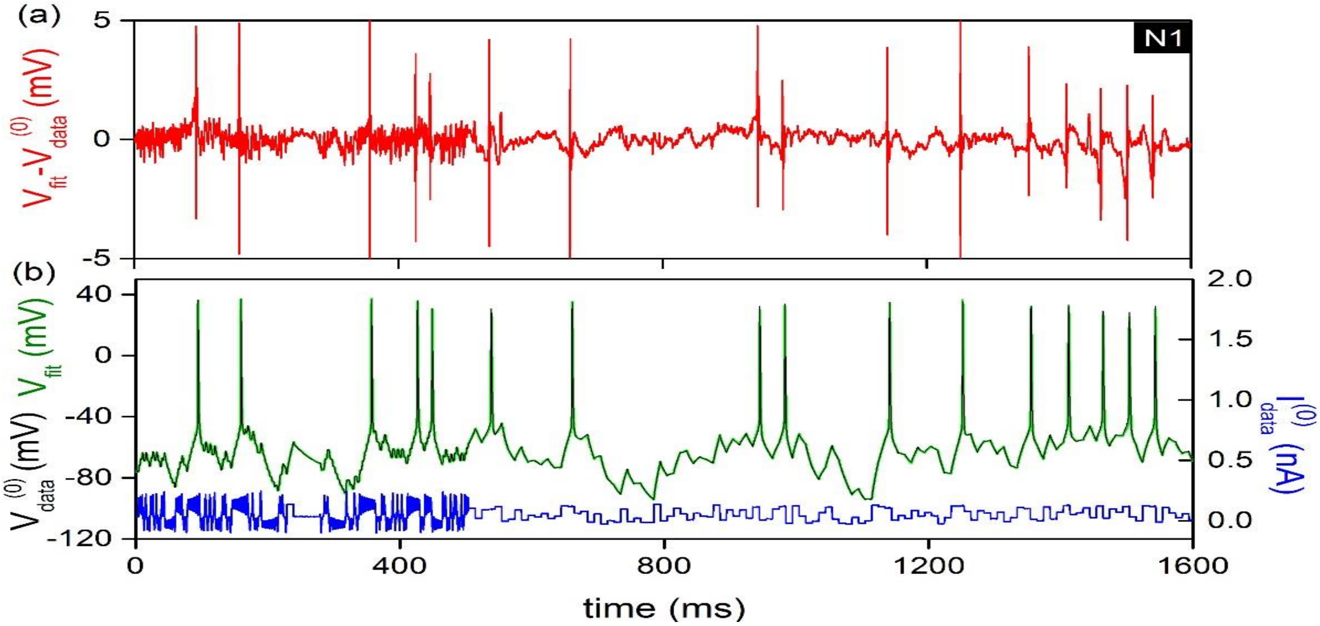

Figure S4: Error made in fitting the output of N1 over the assimilation window.
(a) $V^{*}(t_i)$-$V_{data}(t_i)$ for i=0,2…N. (b) Observed membrane voltage $V_{data}(t_i)$ (black line), current protocol $I_{data}(t_i)$ (green line).

V. Time dependence of gate variables

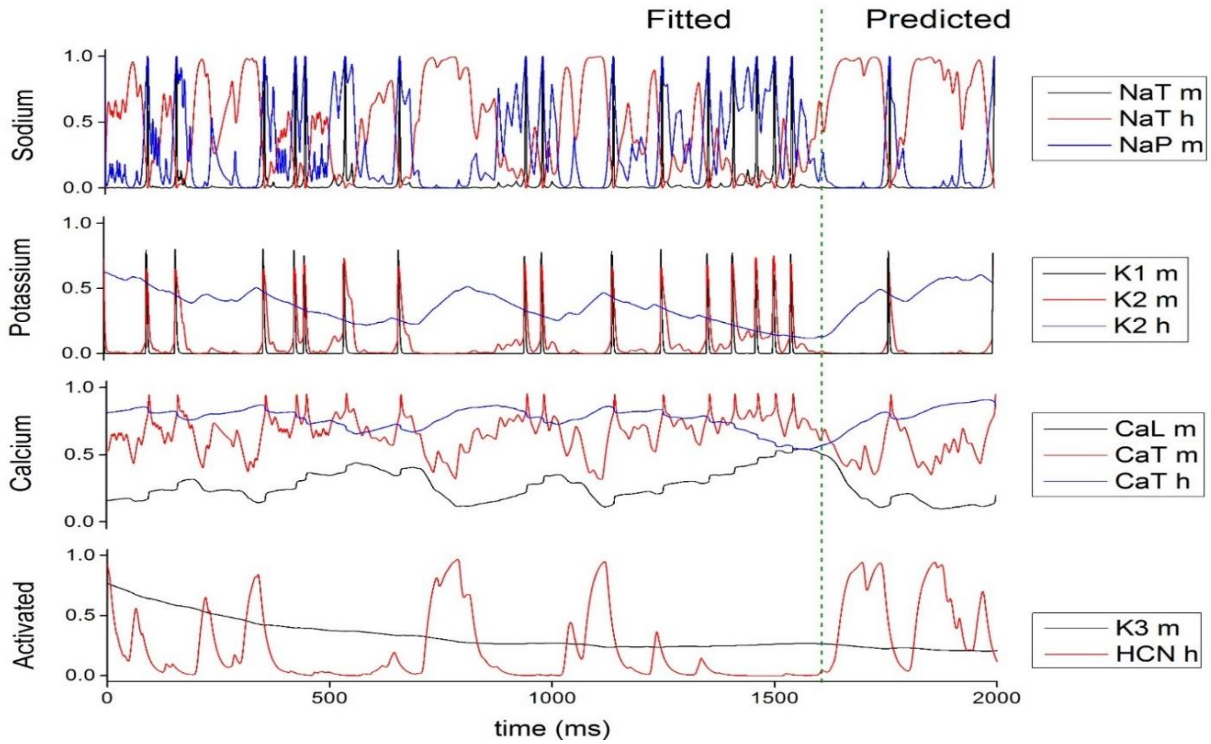

Figure S5: Time evolution of unobserved gate variables of N1 (Epoch 0):
The activation (m) and inactivation (h) gate variables are calculated by minimizing the objective function in the 0-1600ms assimilation window and by forward integration of the completed model from 1600ms-onwards. The fitted range shows the gate variables of the conductance model as the *membrane voltage state variable* is fitted to the measured membrane voltage.

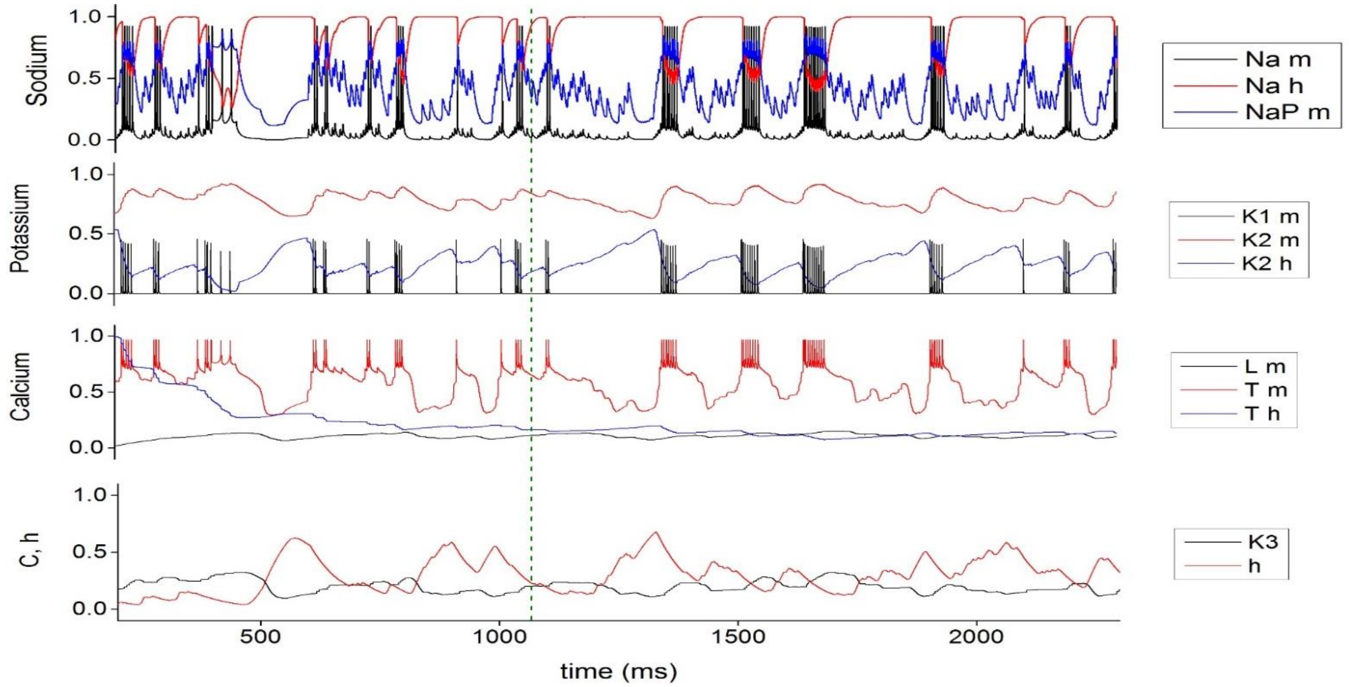

Figure S6: Time evolution of unobserved gate variables of N2 (Epoch 0):
Oscillations in the 190ms - 1190ms assimilation window are calculated by the optimization procedure. Those in the 190ms-1190ms time interval are calculated by forward integration of the completed model from 1190ms onward.

VI. Fitting and prediction of N2 on expanded scale

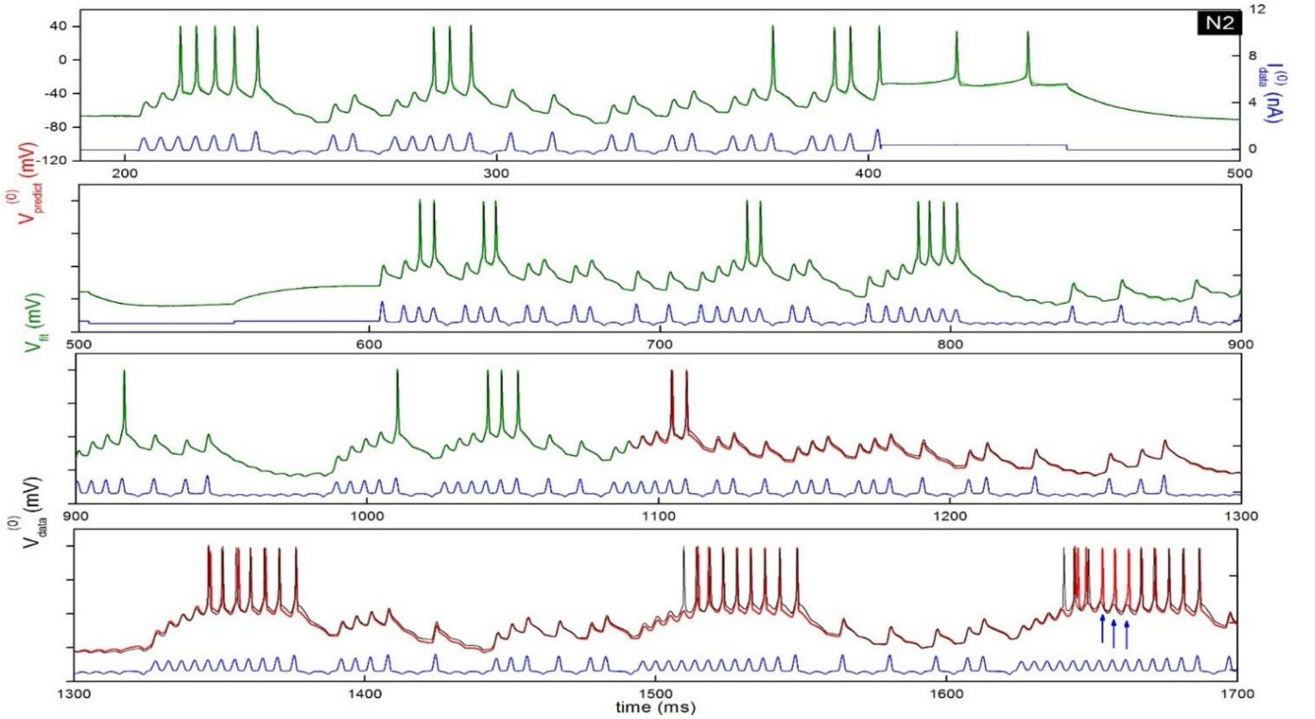

Figure S7: Expanded plot of Fig.2 of the manuscript showing Epoch 0 of Neuron N2:
The arrows at 1653ms, 1657ms and 1662ms indicate three voltage peaks which are correctly predicted by the model (red line) but are absent from the data (black line). This interruption in the bursting pattern is inconsistent with the continuous firing observed in the two earlier bursts at 1360ms and 1530ms under a similarly rising current stimulation. The missing spikes are likely due to internal variability of the HVC neuron. *They indicate that the predictions of the completed model are sufficiently accurate to identify trial-to-trial fluctuations in neuron output.*

VII. 7 of the 84 experimental protocols used to extract a statistical sample of parameters for N1

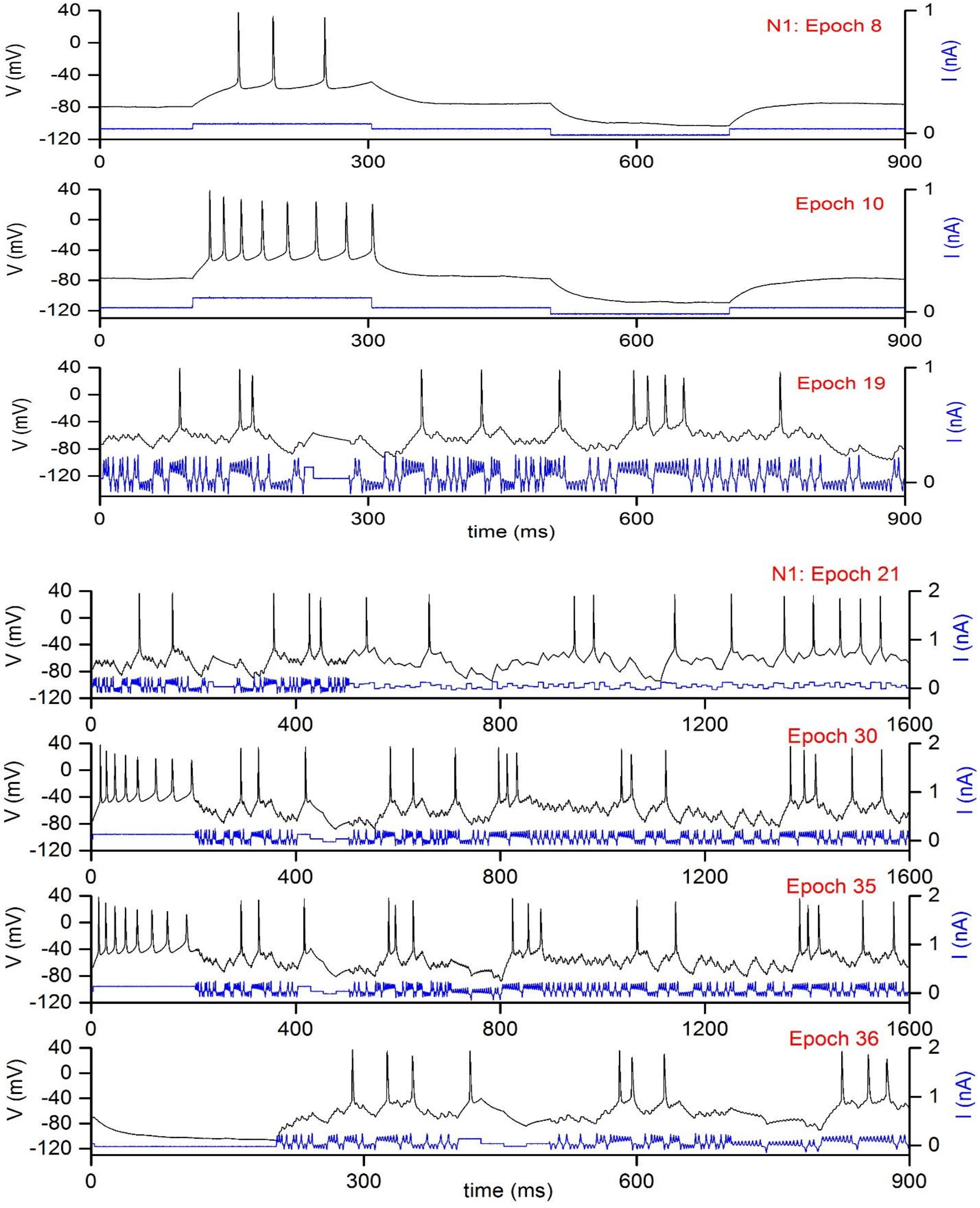

Figure S8: Epochs used to extract different sets of parameters for neuron 1.
The width of each graph is the assimilation window.

VIII. 7 of the 84 protocols used to extract a statistical sample of parameters for N2

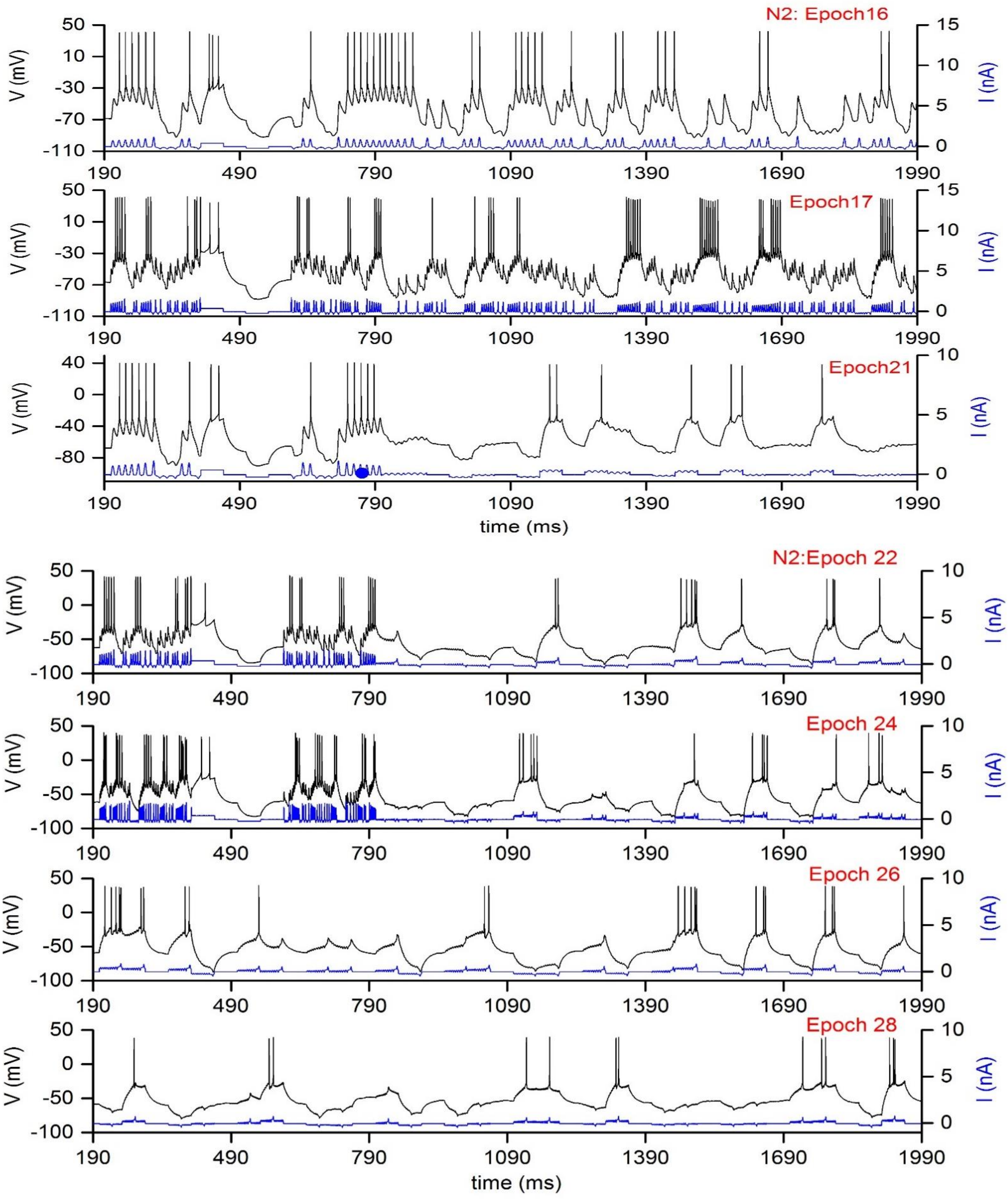

Figure S9: Epochs used to extract different sets of parameters for neuron 2.
The width of each graph (190ms-1990ms) is the assimilation window.

IX. Covariance matrices of N1 and N2

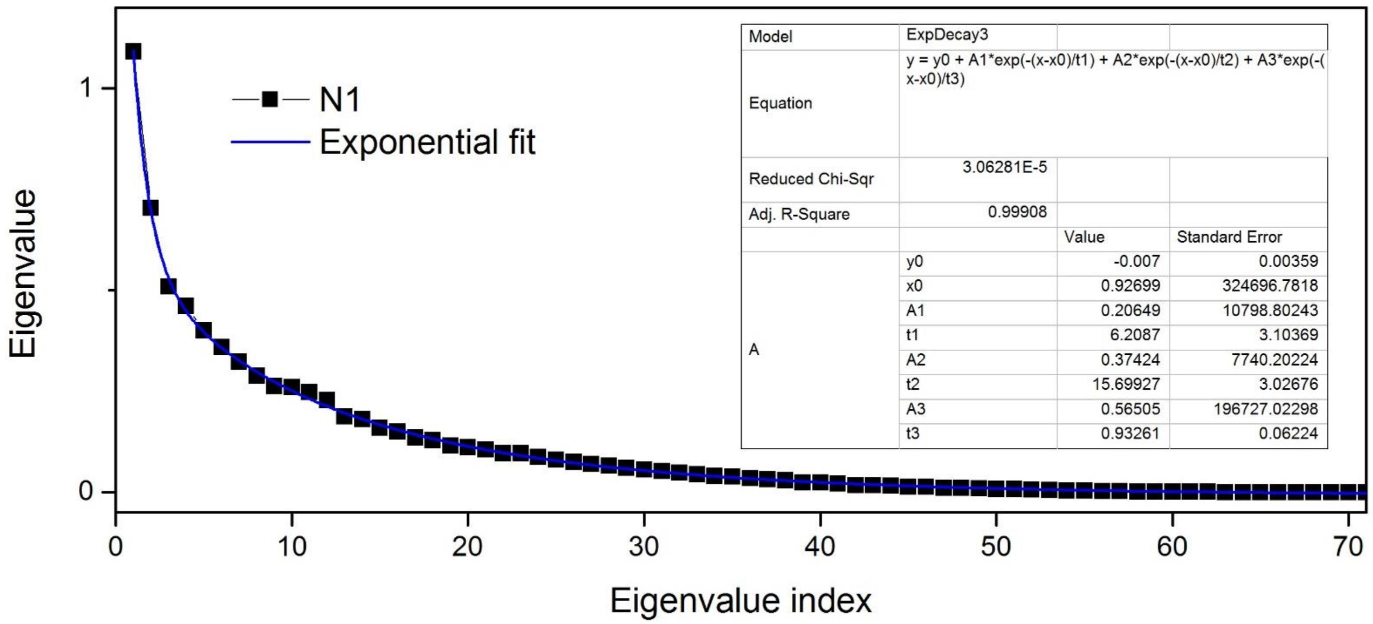

Figure S10: Exponential decay of eigenvalue spectrum
The best fit line (blue line) is a sum of three exponentials with different rates of decay.

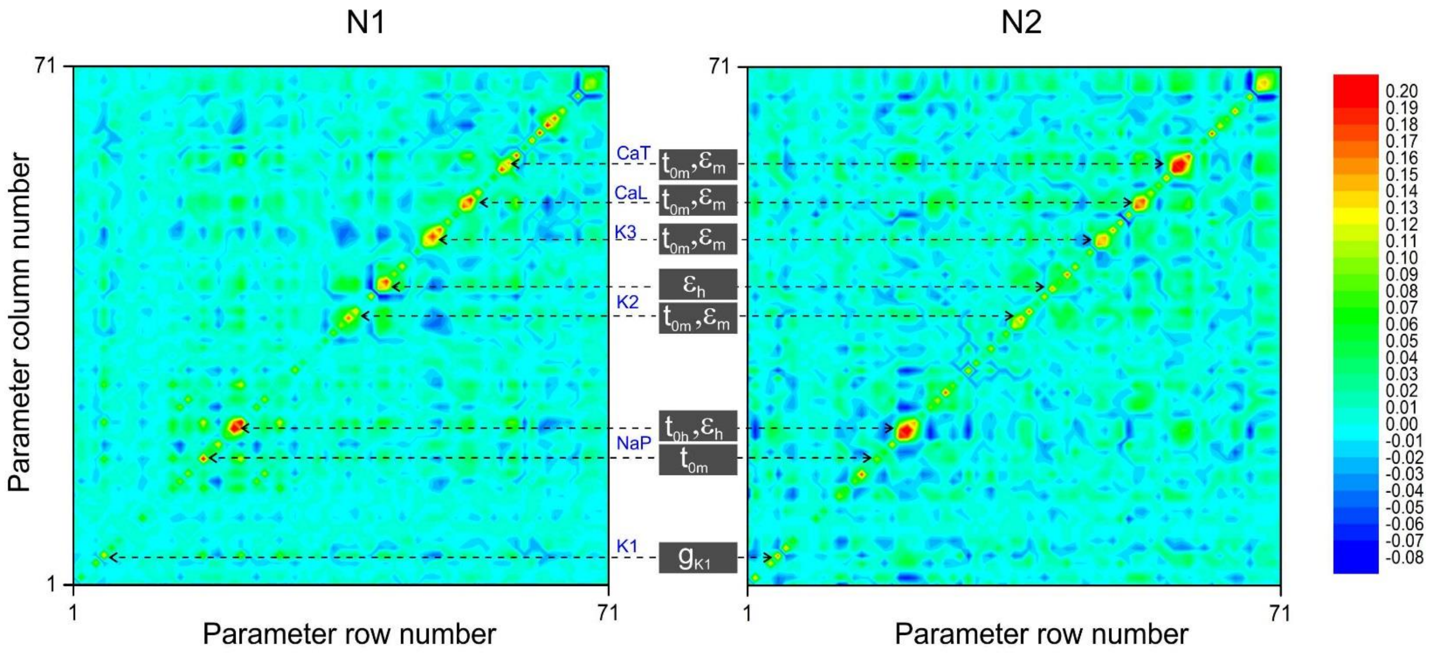

Figure S11: The covariance matrices of neurons N1 and N2
Each covariance matrix was constructed from a statistical sample of 84 sets of parameters extracted over a range of assimilation windows. Parameters were ordered along the rows and columns of matrices in a slightly different order from Table II and as follows:

1. C, 2. g_{Na}, 3. g_{NaP}, 4. E_{Na}, 5. g_{K1}, 6. g_{K2}, 7. g_{K3}, 8. E_K, 9. g_L, 10. E_L, 11. g_{out}, 12. g_{HCN}, 13, A, 14. V_m (NaT), 15. dV_m (NaT), 16. dV_{tm} (NaT), 17. t_{0m} (NaT), 18. ε_m (NaT), 19. V_h (NaT), 20. dV_h (NaT), 21. dV_{th} (NaT), 22. t_{0h} (NaT), 23. ε_h (NaT), 24. V_m (NaP), 25. dV_m (NaP), 26. dV_{tm} (NaP), 27. t_{0m} (NaP), 28. ε_m (NaP), 29. V_m (K1), 30. dV_m (K1), 31. dV_{tm} (K1), 32. t_{0m} (K1), 33. ε_m (K1), 34. V_m (K2), 35. dV_m (K2), 36. dV_{tm} (K2), 37. t_{0m} (K2), 38. ε_m (K2), 39. V_h (K2), 40. dV_h (K2), 41. dV_{th} (K2), 42. t_{0h} (K2), 43. ε_h (K2), 44. δ_h (K2), 45. V_m (K3), 46. dV_m (K3), 47. dV_{tm} (K3), 48. t_{0m} (K3), 49. ε_m (K3), 50. V_h (CaL), 51. dV_h (CaL), 52. dV_{th} (CaL), 53. t_{0h} (CaL), 54. ε_h (CaL), 55. V_m (CaT), 56. dV_m (CaT), 57. dV_{tm} (CaT), 58. t_{0m} (CaT), 59. ε_m (CaT), 60. V_h (CaT), 61. dV_h (CaT), 62. dV_{t1} (CaT), 63. dV_{t2} (CaT), 64. t_{0h} (CaT), 65. ε_h (CaT), 66. V_h (HCN), 67. dV_h (HCN), 68. dV_{th} (HCN), 69. t_{0h} (HCN), 70. ε_h (HCN), 71. ρ.

Finite off-diagonal elements (horizontal and vertical green lines in the covariance matrices) indicate the correlated parameters. Juxtaposing the covariance matrices of N1 and N2, suggests that correlated parameters do not occur at random but happen to be the same in N1 and N2 (horizontal arrows). These are the parameters that define the recovery time constant of ion channels: (NaT) t_{0m} , t_{0h} & ε_h ; (K2) t_{0m} & ε_m , ε_h ;(K3) t_{0m} & ε_m ; (CaL) t_{0m} & ε_m; (CaT) t_{0m} & ε_m and to a lesser extent the conductance of the transient potassium channel: (K1) g_{K1}.

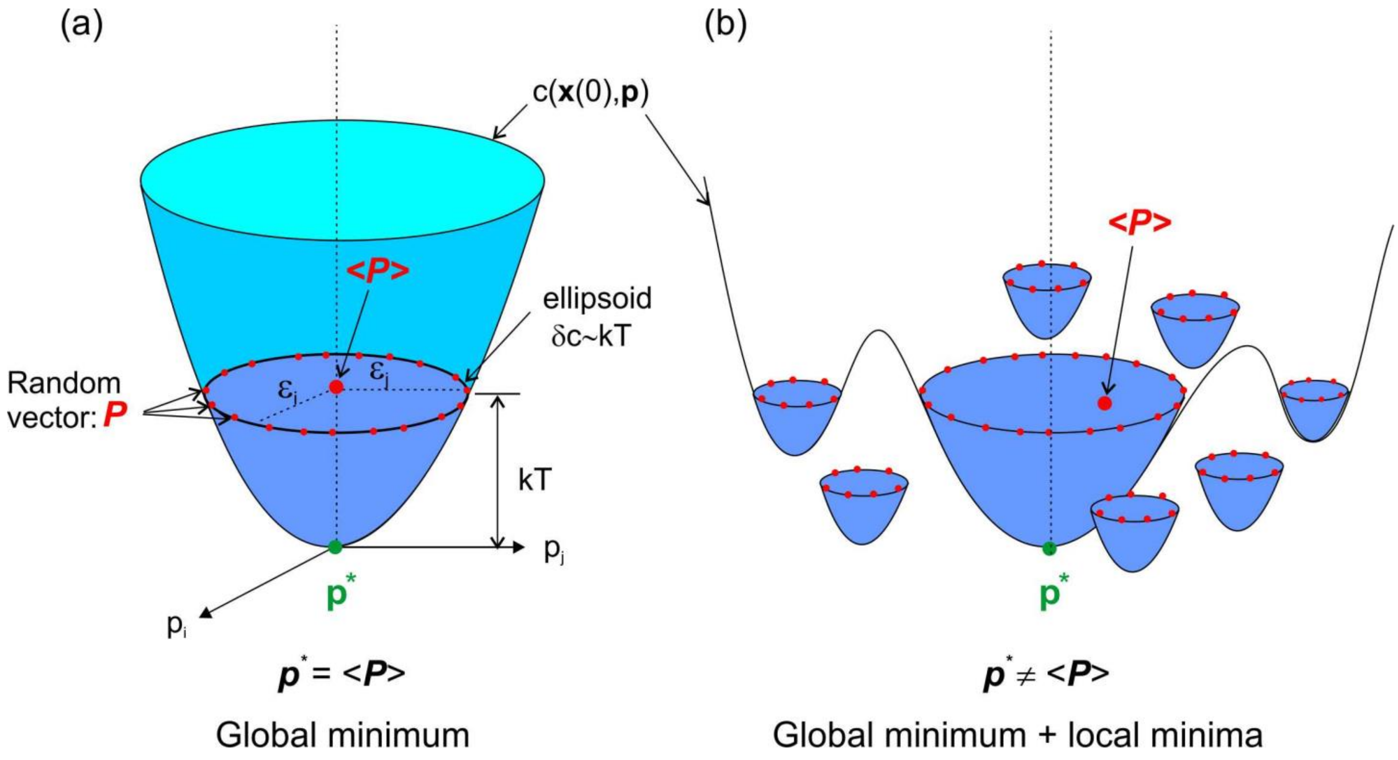

Figure S12: Minimum finding in a noisy environment

Experimental noise has the characteristics of a residual temperature T which prevents reaching the global minimum of the objective function through direct parameter search. Instead, the tip of random vector $\boldsymbol{P}$ lies on the surface of a K-dimensional ellipsoid $\delta c = kT$. Each set of parameters $\boldsymbol{P}$ extracted from one assimilation window gives one point on this surface (red dots). (a) Once a sufficiently large statistical sample of parameters ($R>L$) maps the ellipsoid, the maximum likelihood expectation <$\boldsymbol{P}$> yields the global minimum $\mathbf{p}^*$. The parameter search has converged near the global minimum because the covariance matrices and eigenvalue spectra exhibit identical patterns in N1 and N2. If the parameter search had converged to local minima instead, (b) the covariance matrices of N1 and N2 would have different structures reflecting the different environments around these minima.

Calculation of the entropy of the noise component in the membrane voltage:

Separate the experimental signal $V_{data}(t)$ into the useful signal $V_{use}(t)$ and the noise component $v_n(t)$.

Insert in the cost function:

$$
\begin{aligned}
c(\vec{x}(0),\vec{p}) &= \frac{1}{2}\sum_{i=0}^{i=N}\left(V_{data}(t_i) - V(t_i,\vec{x}(0),\vec{p})\right)^2 \\
&= \frac{1}{2}\sum_{i=0}^{i=N}\left(V_{use}(t_i) + v_n(t_i) - V(t_i,\vec{x}(0),\vec{p})\right)^2 \\
&= \frac{1}{2}\sum_{i=0}^{i=N}\left(V_{use}(t_i) - V(t_i,\vec{x}(0),\vec{p})\right)^2 + \frac{1}{2}\sum_{i=0}^{i=N}\left(v_n(t_i)\right)^2
\end{aligned}
$$

The cross term cancels as it is proportional to the noise average which is zero. The second term on the RHS gives the variance of the membrane voltage which when driven by thermal fluctuations is given by Nyquist theorem:

$$\left\langle v_n^2 \right\rangle = \frac{1}{N+1}\sum_{i=0}^{i=N}\left(v_n(t_i)\right)^2 = 4R\,k_B T\,\Delta f$$

$$c(\vec{x}(0),\vec{p}) = \frac{1}{2}\sum_{i=0}^{i=N}\left(V_{use}(t_i) - V(t_i,\vec{x}(0),\vec{p})\right)^2 + \underbrace{2(N+1)R\,k_B\,\Delta f}_{\text{Entropy}}\;T$$

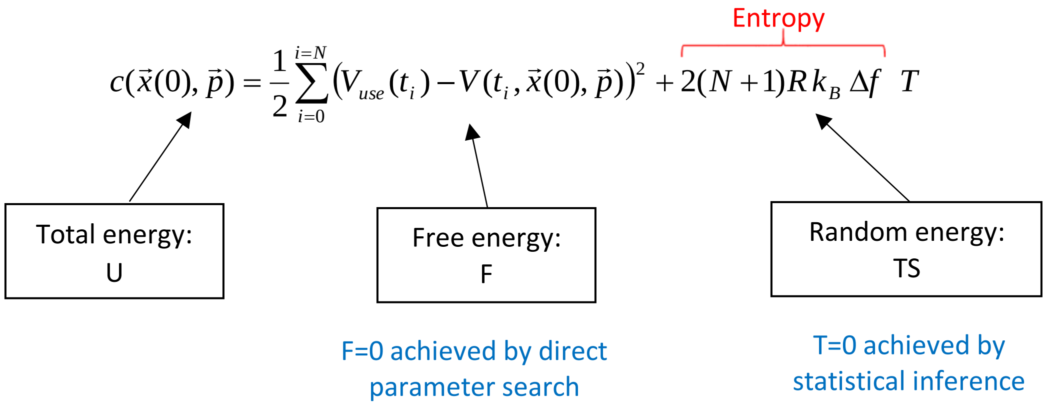

Hence:

$$\delta c = ST$$

Noise behaves as a thermal energy that sets a lower limit to the free energy of the system.

Entropy of the system:	$S = 2(N+1)R\,k_B\,\Delta f$
Resistance of the neuron membrane:	R
Frequency band within which fluctuations are measured:	Δf
Boltzmann's constant:	k_B
